# The miR-25-93-106b cluster regulates tumor metastasis and immune evasion via modulation of CXCL12 and PD-L1

**DOI:** 10.18632/oncotarget.15450

**Published:** 2017-02-17

**Authors:** Michele Cioffi, Sara M Trabulo, Mireia Vallespinos, Deepak Raj, Tony Bou Kheir, Meng-Lay Lin, Julfa Begum, Ann-Marie Baker, Ala Amgheib, Jaimy Saif, Manuel Perez, Joaquim Soriano, Manuel Desco, Maria Victoria Gomez-Gaviro, Lorena Cusso, Diego Megias, Alexandra Aicher, Christopher Heeschen

**Affiliations:** ^1^ Stem Cells & Cancer Group, Spanish National Cancer Research Centre (CNIO), Madrid, Spain; ^2^ Stem Cells in Cancer & Ageing, Barts Cancer Institute, Queen Mary University of London, London, UK; ^3^ Centre for Tumour Biology, Barts Cancer Institute, Queen Mary University of London, London, UK; ^4^ School of Clinical Sciences, University of Bristol, Bristol, UK; ^5^ Confocal Microscopy Unit, Centro Nacional de Investigaciones Oncológicas, Madrid, Spain; ^6^ Departamento de Ingenieria Biomedica e Ingeniería Aeroespacial, Universidad Carlos III de Madrid, Leganés, Spain; ^7^ Instituto de Investigación Sanitaria Gregorio Marañón, Madrid, Spain; ^8^ Centro de Investigación Biomédica en Red de Salud Mental (CIBERSAM), Madrid, Spain

**Keywords:** metastasis, CD274, bone marrow, stromal niche, MDSC

## Abstract

The stromal microenvironment controls response to injury and inflammation, and is also an important determinant of cancer cell behavior. However, our understanding of its modulation by miRNA (miR) and their respective targets is still sparse. Here, we identified the miR-25-93-106b cluster and two new target genes as critical drivers for metastasis and immune evasion of cancer cells. Using miR-25-93-106b knockout mice or antagomiRs, we demonstrated regulation of the production of the chemoattractant CXCL12 controlling bone marrow metastasis. Moreover, we identified the immune checkpoint PD-L1 (CD274) as a novel miR-93/106b target playing a central role in diminishing tumor immunity. Eventually, upregulation of miR-93 and miR-106b via miR-mimics or treatment with an epigenetic reader domain (BET) inhibitor resulted in diminished expression of CXCL12 and PD-L1. These data suggest a potential new therapeutic rationale for use of BET inhibitors for dual targeting of cancers with strong immunosuppressive and metastatic phenotypes.

## INTRODUCTION

The bone marrow (BM) stromal niche harbors and controls hematopoietic stem cells (HSC) [1], and also plays a critical role in the recruitment and survival of normal and neoplastic cells via production of chemoattractants. Still, our understanding of the epigenetic regulation of the stromal niche during homeostasis and in response to injury or tumorigenesis remains very limited [1, 2]. Therefore, we studied the role of microRNA (miR) in regulating the stromal niche. MiR are short, non-coding RNAs regulating target mRNAs. Base pair interactions between miR and target mRNAs within the 3′UTR (untranslated region) result in the degradation of the target mRNAs or modulation of their translation. Using tissue ischemia as a model for activating the BM niche, we found that the miR-25-93-106b cluster was most prominently upregulated encouraging us to validate*in silico* predicted targets of this cluster, i.e. the chemokine CXCL12 (stromal cell-derived factor-1; SDF-1), ligand for the chemokine receptor CXCR4, and the immune modulator CD274 (programmed cell death ligand-1; PD-L1), which binds to CD279 (PD-1).

CXCL12 is a key attraction and retention signal for stem cells including cancer stem cells [3, 4] via activation of its receptor CXCR4. Cells expressing strongly CXCL12 in the stromal niche are mostly endothelial cells and perivascular mesenchymal stromal cell populations including cancer-associated fibroblasts [5, 6], and CXCL12 levels are variably modulated in response to local or remote pro-inflammatory stimuli [7–9]. The PD-L1 – PD-1 signaling pathway efficiently inhibits T-cell activation [10, 11] and growing evidence demonstrates that blockade of PD-1 or its ligand PD-L1 significantly enhances anti-tumor immunity resulting in durable tumor regression in a sizable fraction of patients with advanced cancers [12]. Therefore, advancing our understanding of the underlying regulatory mechanisms for these two critical pathways may also provide the basis for the development of more efficient cancer treatments.

## RESULTS

### Upregulation of the miR-25-93-106b cluster in the BM stromal niche in response to remote tissue insult

**Figure 1:**
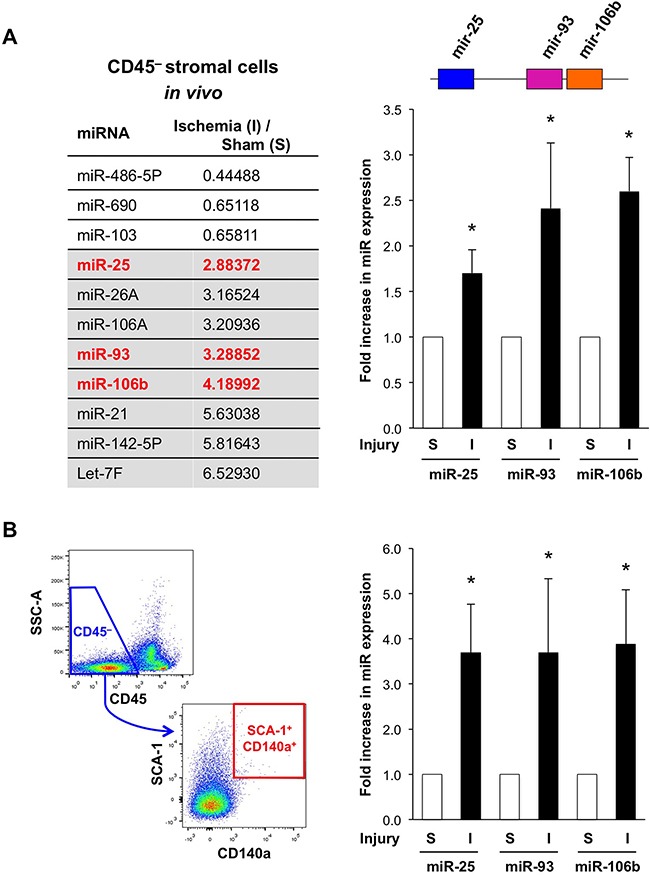
Ischemia-induced up-regulation of miR-25-93-106b in the bone marrow (BM) stromal niche **A**. MiRNA array for CD45^–^ BM stromal cells following sham surgery (S) or ischemia induction (I). **Grey background**: most prominently upregulated miR, **red**: members of the miR-25-93-106b cluster (**left**). Validation by qRT-PCR; n=5-6, * p<0.05 (**right panel**). **B**. Gating strategy (**left**) and quantification (**right**) of CD45^–^CD140a^+^SCA-1^+^ mesenchymal progenitor cells. Quantification by qRT-PCR; n=3-4, * p<0.05.

To study the role of miR in the regulation of the stromal niche, we examined changes in miR expression in BM stromal cells in response to tissue insult (Figure 1A). Considering that many cancers are poorly vascularized and invested with inflammation [13], we used two reproducible and hypothesis-generating model systems, unilateral hind limb ischemia and total body irradiation (TBI), which can also be applied to respective knockout mice in a timely fashion. First, we analyzed the BM stroma in the contralateral, non-ischemic hind limb of the hind limb ischemia model (Supplementary Figure 1A–1D). We found all three members of the miR-25-93-106b cluster to be consistently increased (Figure 1A). Upregulation of miR-25, 93, and 106b was confirmed by qRT-PCR in sorted CD45^–^ BM cells and CD45^–^CD140a^+^SCA-1^+^ mesenchymal progenitor cells, respectively (Figure 1B) [14]. In line with the hypothesis that miR-25-93-106b is important for tissue regeneration, induction of hind limb ischemia or myocardial infarction in miR-25-93-106b KO mice resulted in a significantly reduced limb perfusion and larger infarct sizes, respectively (Supplementary Figure 1A-1E). In addition, miR-25-93-106b KO mice undergoing myocardial infarction showed a strong desmoplastic response in line with an increased fibroblastoid colony-forming activity detected in miR-25-93-106b KO mice (Supplementary Figure 4). Consistently, in pancreatic tumors as a prototypic cancer with extensive desmoplasia, we also found a suppression of the miR-25-93-106b cluster in stromal cells relative to the cancer cells (Supplementary Figure 2A). These data were also further confirmed by assessment of freshly isolated and sorted stromal and cancer cells by qRT-PCR showing lower expression of miR-93 and miR-106b in stromal cells than cancer cells (Supplementary Figure 10). In addition, we performed in situ hybridization (ISH) for miR-106b visualizing miR-106b expression in primary pancreatic cancer and liver metastasis, thereby confirming expression in both stromal cells and cancer cells as well as inverse target regulation (Supplementary Figure 11).

### Enhanced recruitment and invasion of bone marrow cells upon downregulation of miR-93/106b in the stromal niche

**Figure 2:**
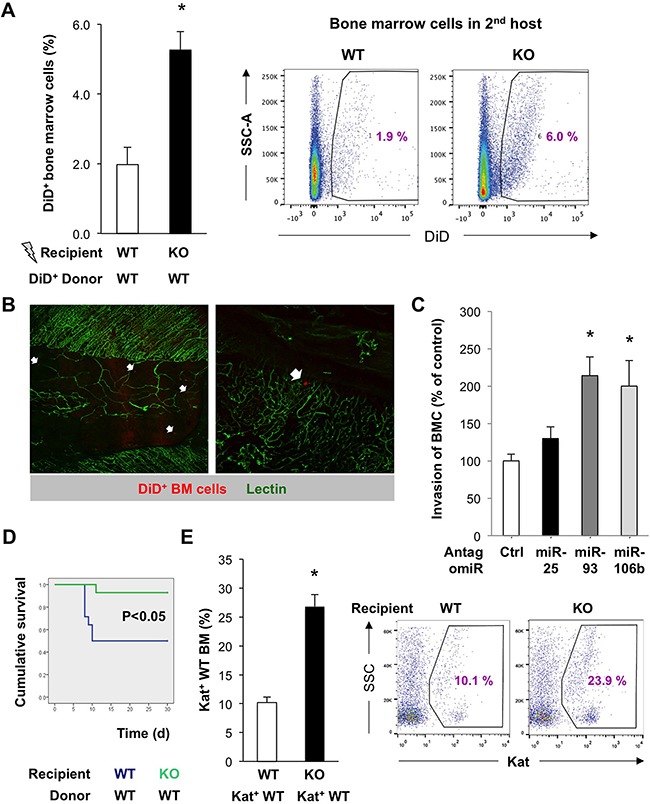
Enhanced recruitment and invasion of bone marrow cells upon downregulation of miR-93/106b in the stromal niche **A**. *In vivo* homing of DiD-labeled BMC to the irradiated BM of WT vs miR-25-93-106b KO mice, n=4, * p<0.05 (**left**). Representative flow cytometry (**right**). **B**. *Ex vivo* confocal analysis of irradiated WT sternal BM 48 hours after i.v. injection of DiD^+^ BM cells (red). Vascularization is identified by lectin staining (green). Homing of individual DiD^+^ BM cells is indicated (arrows). **C**. *In vitro* invasion of BMC towards WT-MSC in the presence or absence of miR-25/93/106b antagomiR. n=7, * p<0.05 for miR-25/93/106b antagomir treatment versus control antagomir. **D**. Kaplan-Meier survival curves of recipient WT vs KO mice following WT BM transplantation; n=14, * p<0.05. **E**. Homing of Kat^+^ WT BM cells to the BM of irradiated WT vs KO mice; n=3, * p<0.05 (**left**). Representative flow cytometry is shown (**right**).

**Figure 5:**
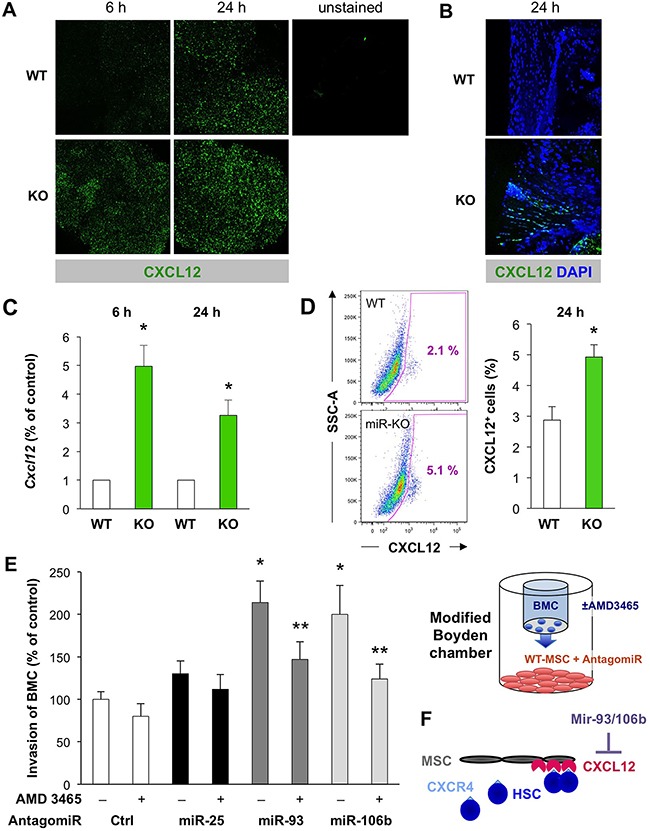
The miR-25-93-106b cluster is a negative regulator of *Cxcl12* in the bone marrow stromal niche **A**. *Ex vivo* confocal microscopy analysis for CXCL12 expression in WT vs miR-25-93-106b KO BM cells following *in vivo* whole body irradiation. Representative images were taken 6 and 24 hours after irradiation. **B**. High-power confocal microscopy of CXCL12 expression in the BM stromal niche *ex vivo*. **C**. *Cxcl12* mRNA expression was quantified by qRT-PCR 6 and 24 hours after irradiation; n=5, * p<0.05. **D**. Representative flow cytometry of intracellular CXCL12 expression in WT vs KO BM (**left**) and quantification; n=3, * p<0.05 (**right**). **E**. *In vitro* invasion of BMC towards WT-MSC in the presence or absence of miR-25/93/106b antagomiR. The CXCR4 antagonist AMD 3465 was used to inhibit CXCL12/CXCR4 signaling. Control groups are replicated from Figure 2C; n=7, * p<0.05 for miR-25/93/106b antagomir treatment versus control antagomir, ** p<0.05 for CXCR4 antagonist treatment versus PBS control. Quantification **(left)** and schematic illustration **(right)**. **F**. Role of the miR-25-93-106b cluster in the bone marrow niche. Illustration that the bone marrow niche in our *in vitro* setup is mimicked by MSC expressing CXCL12.

Tissue repair and tumor development are accompanied by the influx of various cells including BM cells (BMC). We used DiD-labeled HSC-containing BMC freshly derived from WT mice to study their capacity to home to the BM of irradiated miR-25-93-106b KO vs. WT mice (Figure 2A/2B). We observed that DiD^+^ WT BMC were more efficiently recruited to the BM stroma of miR-25-93-106b KO mice as compared to WT BM suggesting that miR-25-93-106b KO mice produce higher levels of chemoattractants following tissue insult, i.e. total body irradiation (TBI). To validate individual cluster members as crucial for the observed phenotype, we studied the invasion of WT BMC towards CD45^–^ WT BM-derived mesenchymal stem cells (WT-MSC) that were pre-treated with control or antagomiR for miR-25, 93, and 106b. We found enhanced invasion/migration through the Matrigel™ layer for WT-MSC treated with antagomiR for miR-93 and 106b, but not for miR-25 (Figure 2C/Figure 5E).

To further corroborate these findings, we used a distinct setting where mice were exposed to TBI followed by BM transplantations using low numbers (10^5^) of donor Katushka^+^ WT BMC so that successful engraftment was critically dependent on a proficient BM niche. As opposed to remote tissue ischemia, TBI suppressed the expression of the miR-25-93-106b cluster (Supplementary Figure 2B). Survival of mice following WT BM transplantation was almost two-fold higher for miR-25-93-106b KO recipients as compared to WT recipients (Figure 2D). This was in line with increased numbers of Katushka^+^ WT BM cells in the BM of miR-25-93-106b KO recipient mice (Figure 2E) suggesting that more WT BM cells had migrated to the TBI-injured BM of miR-25-93-106b KO mice. In line with these data, WT CD45^+^C-KIT^+^ BMC showed enhanced invasiveness when migrating towards adherently growing miR-25-93-106b KO MSC relative to WT MSC cell layers (Supplementary Figure 2C).

Reverse experiments using miR-25-93-106b KO BM cells transplanted into WT recipients resulted in impaired survival of the mice as compared to WT mice transplanted with WT BM cells (Supplementary Figure 3A). The impaired BM reconstitution by KO BM could also be demonstrated by reduced levels of platelet-producing megakaryocytes and neutrophilic granulocytes resulting in prolonged bleeding times and neutropenia (Supplementary Figure 3B) as well as reduced levels of granulocyte and megakaryocyte progenitors in *in vitro* colony-forming unit (CFU) formation assays (Supplementary Figure 3C). Interestingly, while myeloid progenitors were also reduced, fibroblast progenitors assessed as fibroblastoid CFU (CFU-F) were increased in the BM of miR-25-93-106b KO mice, which may at least in part rationalize the enhanced desmoplastic response in these mice (Supplementary Figure 1E) that is also characteristic of many tumors [13]. Consistently, fibroblast activation protein (FAP)^+^CD45^–^ fibroblasts and FAP^+^CD45^+^ fibrocytes were both increased in the BM and peripheral blood of miR-25-93-106b KO mice (Supplementary Figure 4A/4B). About 60% of CD45^+^ colony-derived cells were CD11b^+^, of which ∼3% were FAP^+^. Importantly, while peripheral blood-derived fibroblastoid CFU from WT mice exhibited increased numbers of CD45^–^C-KIT^+^ vascular progenitors in response to ischemia, miR-25-93-106b KO mice showed impaired mobilization of vascular progenitors into the peripheral blood (Supplementary Figure 4C).

### Enhanced recruitment and invasion of cancer cells upon downregulation of miR-93/106b

**Figure 3:**
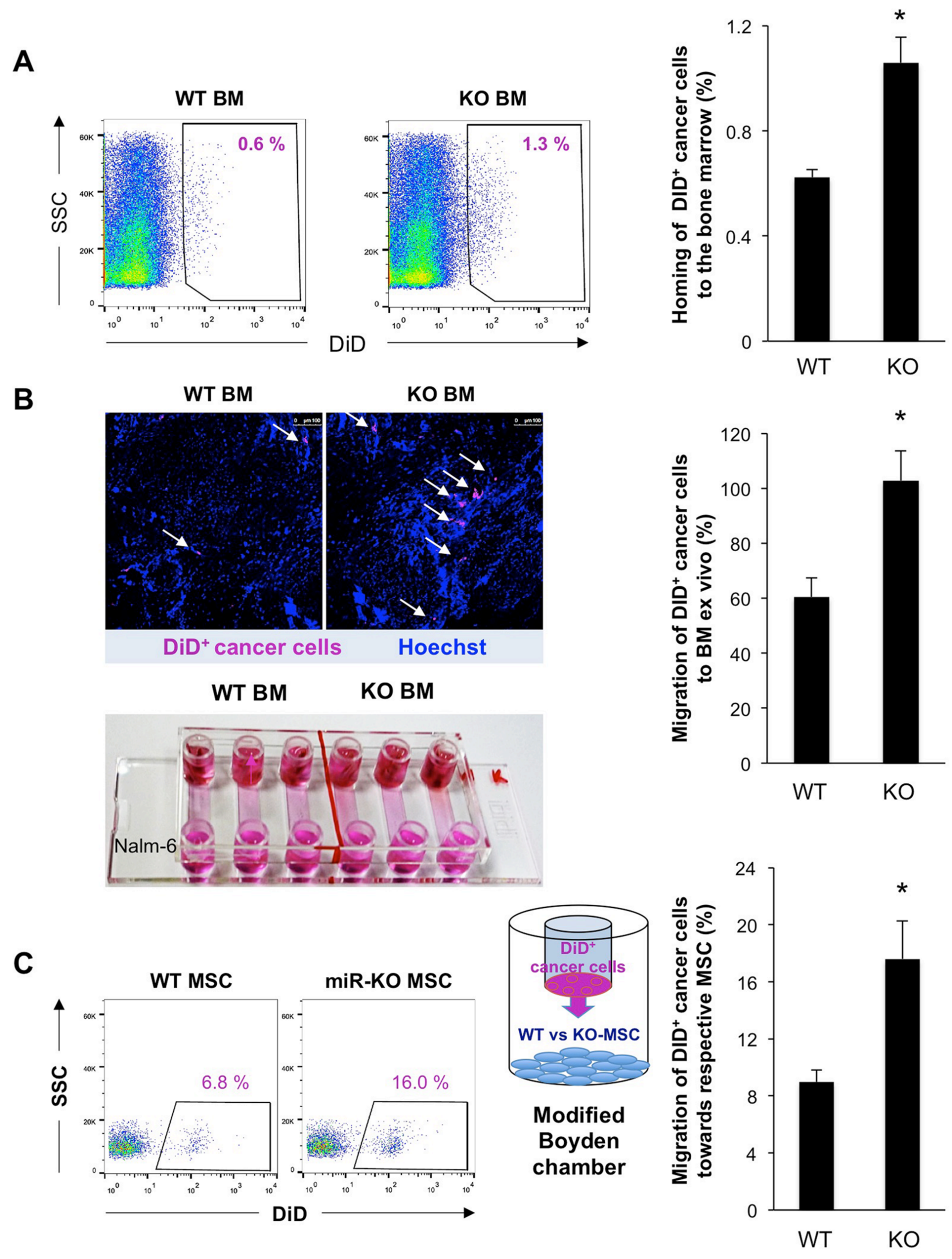
Enhanced recruitment and invasion of cancer cells upon downregulation of miR-93/106b in the stromal niche **A**. Number of DiD^+^ Nalm-6 cells found in the BM of WT vs KO mice 6 hours after injection; n=4, * p<0.05. Representative flow cytometry **(left)** and quantification **(right)**. **B**. Migration of DiD^+^ Nalm-6 cells to WT and KO BM *ex vivo*; n=6, * p<0.05. Representative confocal image (**upper left**), migration chamber (**lower left**), quantification (**right**). **C**. Migration/invasion of DiD^+^ Nalm-6 cells to WT and KO MSC following irradiation; n=4, * p<0.05.

To validate these findings in the context of cancer metastasis, we next studied the homing of human Nalm-6 cancer cells to the BM as a crucial step in metastasis, which still represents a primary reason for cancer-associated lethality. DiD-labeled Nalm-6 cells were intravenously injected into irradiated miR-25-93-106b KO and WT mice, respectively, and the BM was examined 6 hours afterward showing increased homing to MiR-25-93-106b KO BM (Figure 3A). These data were confirmed by *ex vivo* migration assays employing explanted and slit bones of irradiated miR-25-93-106b KO or WT mice, placed at one end of a flow chamber, while DiD^+^ Nalm-6 cells were placed at the other end, both connected via a microfluidic channel. We found increased numbers of Nalm-6 cells migrating towards miR-25-93-106b KO bone fragments (Figure 3B). DiD^+^ Nalm-6 cells also migrated more efficiently towards irradiated miR-25-93-106b KO BM-derived MSC as compared to WT MSCs (Figure 3C).

### Identification of potentially relevant targets for the miR-25-93-106b cluster

**Figure 4:**
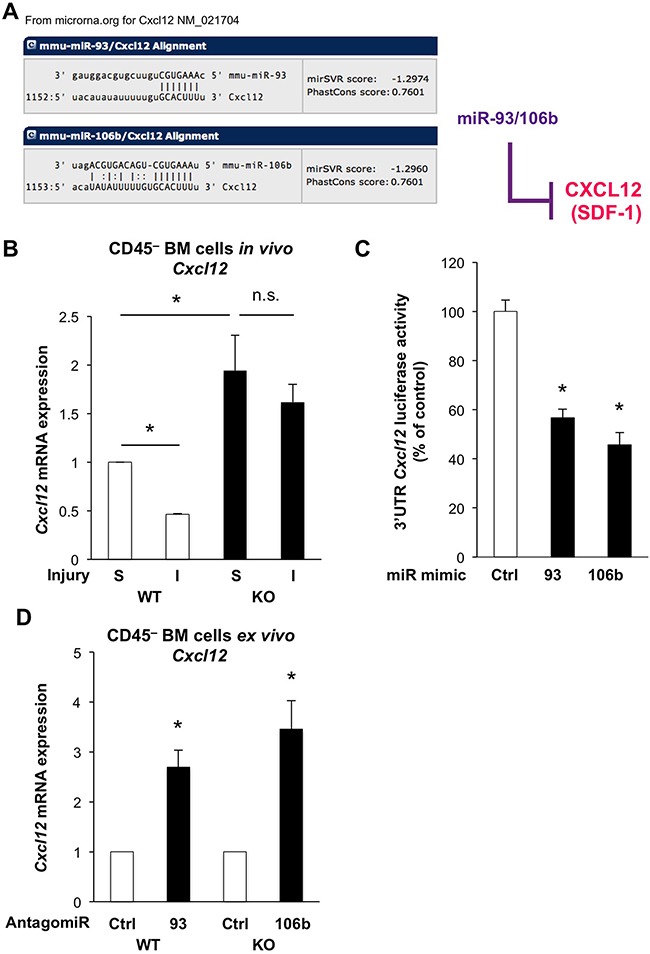
The miR-25-93-106b cluster is a negative regulator of *Cxcl12* **A**. *In silico* prediction of *Cxcl12* as miR-93/106b target by www.microrna.org. **B**. *Cxcl12* expression of CD45^–^BM cells in WT vs miR-25-93-106b KO mice following sham surgery (S) or induction of ischemia (I); n=3, * p<0.05. **C**. 3′UTR *Cxcl12* luciferase assay in HEK293 cells in the presence or absence of miR-93 and miR-106 mimics; n=9, * p<0.05. **D**. *Cxcl12* expression in *ex vivo* CD45^–^ BM cells with or without miR-93/106b antagomiR; n=5, * p<0.05.

**Figure 6:**
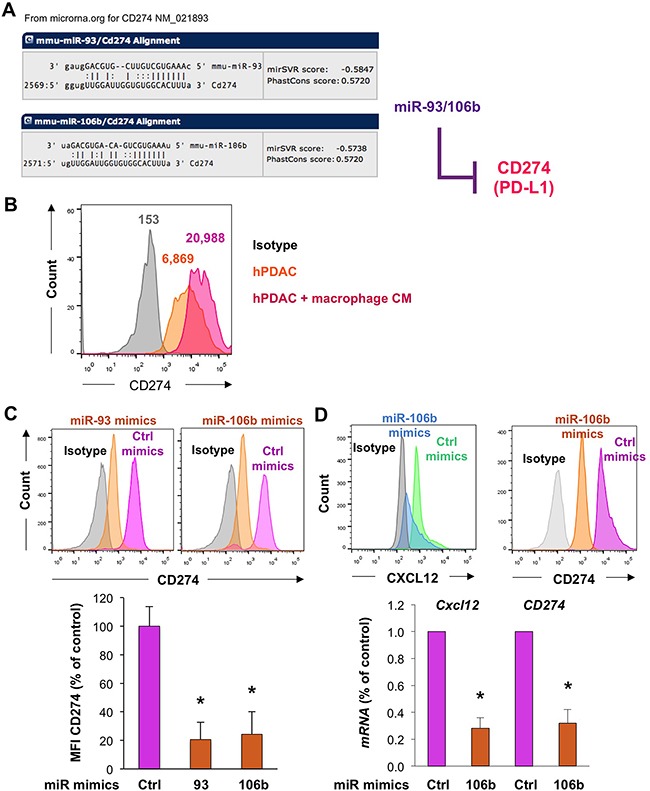
Regulation of the miR-25-93-106b target CD274 in pancreatic cancer cells **A**. *In silico* prediction of CD274 as a target of miR-93 and miR-106b. **B**. Expression of CD274 in pancreatic cancer cells in the absence or presence of macrophage-conditioned medium (CM). **C**. CD274 expression as determined by flow cytometry in freshly isolated murine primary PDAC tumors in the presence of miR-93 or miR-106b mimics 20 hours after transfection; n=3, * p<0.05. **D**. Dual inhibition of *Cxcl12* and *CD274* expression in freshly isolated murine primary PDAC tumors as determined by flow cytometry and qRT-PCR, n=4-5, * p<0.05.

**Figure 7:**
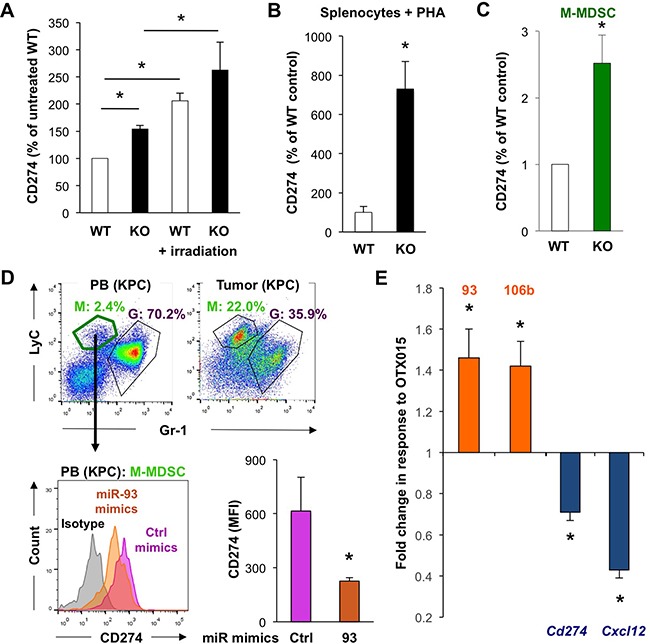
MiR-93/106b regulates CD274 and CXCL12 expression **A**. CD274 expression in CD11b^+^ BM cells as determined by flow cytometry 6 hours following total body irradiation. Quantification of data with untreated WT set as 100%; n=3-5, * p<0.05. **B**. Expression of CD274 in splenocytes following PHA activation; n=4, * p<0.05. **C**. Quantification of CD274 expression in sorted M-MDSC derived from WT vs miR-25-93-106b KO mice using qRT-PCR; n=4. **D**. MiR-93 mimics regulate CD274 expression in M-MDSC of KPC mice. Representative flow cytometry for M-MDSC (green) and G-MDSC (purple) in the peripheral blood (PB) and pancreatic tumor (KPC) **(upper)**. Sorted M-MDSC from PB were treated *ex vivo* with control or miR-93 mimics and analyzed for the expression of CD274. Representative flow cytometry (**lower left**) and quantification (**lower right**); n=3, *p<0.05. **E**. Effect of OTX015 (500nM; 72 hours) on the target genes cd274 and cxcl12 in myeloid and fibroblastoid stromal cells, n=6, * p<0.05.

To interrogate the large list of potentially relevant targets modulated by miR-25-93-106b, we isolated CD45^–^ BM stromal cells from miR-25-93-106b KO vs. WT mice following induction of ischemia for 48 hours. We did not only study established targets of the mir-25-93-106b cluster i.e. *p21* [15], integrin b8 *(Itgb8)* [16], and transforming growth factor b receptor II (*Tgfbr2*) [17], but also *in silico* predicted targets such as *Zeb2* (Supplementary Figure 5), *Cxcl12* (Figure 4A), and *Cd274* (Figure 6A). Not unexpectedly, all above target genes, except *Cd274* (Figure 7A), were downregulated in WT mice in response to tissue insult but were unleashed in miR-25-93-106b KO mice (Figure 4B & Supplementary Figure 5). For further investigation we selected CXCL12 as an established critical regulator of stem cell homing and mobilization, respectively [18, 19] and PD-L1 and PD1 as a crucial immune checkpoint with strong translational relevance.

### The miR-25-93-106b cluster is a negative regulator of CXCL12

Using 3′UTR *Cxcl12* luciferase assays, we first validated that miR-93-5P and miR-106b-5P suppressed luciferase activity establishing *Cxcl12* as a miR-93-5P/106b-5P target (Figure 4C). Moreover, *Cxcl12* was downregulated in CD45^–^ BM stromal cells of WT mice following tissue ischemia, while miR-25-93-106b KO mice showed higher baseline levels for *Cxcl12* in sham-treated mice and lack of suppression following tissue ischemia (Figure 4B). These *in vivo* results were corroborated using CD45^–^ BM cells treated *ex vivo* with miR-93/106b antagomiR demonstrating upregulation of *Cxcl12* (Figure 4D). Again we pursued TBI as a distinct modality of tissue injury and analyzed the bone marrow after 6 and 24 hours for the expression of CXCL12 using confocal microscopy (Figure 5A/5B). During 24 hours, TBI-induced expression of CXCL12 protein in WT BM stromal niches, but CXCL12 levels were significantly higher in miR-25-93-106b KO mice. This was confirmed by qRT-PCR analysis (Figure 5C) and intracellular flow cytometry for CXCL12 (Figure 5D). To functionally validate CXCL12 as a chemokine that is crucial for the observed phenotype, we next studied WT BMC invading and migrating through a Matrigel™ layer towards adherently growing CD45^–^ BM-derived mesenchymal stem cells (MSC). The specific CXCR4 antagonist AMD3465 abrogated the enhanced invasiveness (Figure 5E) demonstrating that the miR-25-93-106b cluster regulates stem cell trafficking via CXCL12 (Figure 5F). In addition, we also corroborated the role of CXCL12 using CRISPR/Cas9-mediated gene editing to create CXCL12 knock-out cells confirming the role for CXCL12 in miR deficiency–induced migration (Supplementary Figure 8).

### The miR-25-93-106b cluster targets CD274 in cancer cells

Next we investigated whether the miR-25-93-106b cluster is also involved in modulating immune tolerance, which is particularly important during tumor cell trafficking and metastasis when cancer cells are directly exposed without a protective tumor microenvironment. Our *in silico* prediction screen suggested that miR-106b-5P and miR-93-5P are targeting the checkpoint inhibitor *Cd274* (PD-L1) (Figure 6A), but not any of the other key players expressed on T-cells that induce tolerance such as *Pdcd-1, Ctla4*, *Ido1/2*, *Lag3, Tim3, Fasl*, and *Cd80/86* (data not shown). In line with this prediction, primary cancer cells also expressed CD274, which was further enhanced by inducing a more invasive phenotype using macrophage-conditioned medium (Figure 6B) [20]. Surprisingly, however, expression of CD274 was lower in the contained subset of cancer stem cells as compared to their more differentiated progenies (Supplementary Figure 6A), whereas miR-106b-5P expression was inversely increased in cancer stem cells (Supplementary Figure 6B); findings that are still in line with previous findings for cholangiocarcinoma CSC [21]. Importantly, expression of CD274 was reduced in bulk cancer cells following treatment with miR-106b-5P and miR-93-5P mimics, respectively (Figure 6C). Intriguingly, dual inhibition of both CXCL12 and CD274 at the mRNA and protein level could be achieved by miR-106b-5P mimics (Figure 6D). Inversely, CD274 expression was enhanced by treatment with miR-106b-5P antagomiR (Supplementary Figure 6C).

### The miR-25-93-106b cluster targets CD274 in stromal cells

In line with above findings for primary cancer cells, we also found that miR-25-93-106b KO mice bear 50% more CD274+ myeloid CD11b^+^ BM cells as compared to WT mice (Figure 7A & Supplementary Figure 7A/7B) and the increase is even further enhanced upon TBI (which reduces expression of miR-25-93-106b). Similar or even greater differences were observed for the analysis of freshly isolated untreated splenocytes (Supplementary Figure 7C), splenocytes stimulated with phytohemagglutinin (PHA) to activate T cells to secrete IFNg as a classical inducer of PD-L1 [22] (Figure 7B), and monocytic myeloid-derived suppressor cells (M-MDSC) (Figure 7C & Supplementary Figure 7D). Sorted CD274-expressing M-MDSC from the peripheral blood of tumor-bearing mice showed a diminished expression of CD274 following treatment with miR-93 mimics (Figure 7D). In line with these findings, freshly isolated tumor cells treated with miR-93-5P and miR-106b-5P mimics also exhibited significantly reduced expression of CD274 (Figure 6C). Intriguingly, these effects could be mimicked by treatment with inhibition of the bromodomain and extraterminal (BET) family of proteins, i.e. using OTX015, which resulted in upregulation of both miRs and subsequent suppression of *Cd274* and *Cxcl12* (Figure 7E). Similar data were obtained for OTX015 and the expression of *Cd274* in primary cancer cells (Supplementary Figure 6D). In addition, we further confirmed the role of the miR-25-93-106b cluster for the regulation of CD274 demonstrating that miR-106b deficiency partially prevented the OTX015-induced suppression of CD274 (Supplementary Figure 9). Therefore, our data demonstrate that miR-93-5P and miR-106b-5P negatively regulate CXCL12 and CD274 expression in both cancer cells and MDSC, and that BET inhibitors represent a suitable upstream modality to control expression of CXCL12 and CD274 via transcriptional regulation of the miR-25-93-106b cluster.

## DISCUSSION

The BM stromal niche plays a crucial role for survival, maintenance, and mobilization of stem and progenitor cells as well as cancer (stem) cells via the production of chemoattractsignals such as CXCL12. Our results elucidate how danger signals such as remote ischemia and systemic total body radio-injury mediate signals to the stromal niche through the evolutionary conserved mir-25-93-106b cluster. Specifically, we identified that the cluster members miR-93 and 106b are essential regulators of the BM stromal compartment for cell homing and mobilization, respectively, through targeting CXCL12. Our data were functionally validated by transplanting normal BM cells into secondary hosts, but interestingly, we also found that the mir-25-93-106b cluster altered the receptiveness of the BM for metastatic cancer cells via the same mechanism. Moreover, we provide conclusive evidence that the immune modulator CD274 is another novel target of miR-93/106b, not only in BM (stromal) cells but also in M-MDSC and primary cancer cells. Thus, our data suggest that miR-93/106b mimics or their transcriptional modulation by bromodomain inhibition could be used for dual targeting of cancers with a strong immunosuppressive and metastatic phenotype.

To date, few miR such as miR-23a and miR-886-3p have been implicated in the regulation of CXCL12 in BM stromal cells *in vitro* [23, 24]. Also, cancer cells and cancer-associated stromal cells showed down-regulation of miR-126, thus enhancing the release of CXCL12 and subsequently recruiting MSCs to further promote tumor cell invasion and metastasis [25]. Importantly, we found that the actual response of the bone marrow stromal niche was dependent on the type of insult. In response to the induction of remote tissue injury, i.e. induction of ischemia, as found in many cancers, we observed members of the miR-25-93-106b cluster to be strongly upregulated resulting in downregulation of CXCL12 in the BM stromal niche. The diminishment of the CXCL12 gradient in the BM niche resulted in the release of stem and progenitor cells and their subsequent mobilization into the peripheral blood and traveling to the site of injury [8]. In contrast, ionizing radiation-induced oxidative stress had previously been demonstrated to alter miR in a time- and dose-dependent manner [26, 27]. Specifically, expression of miR-106b was downregulated in response to higher doses of irradiation [27]. Moreover, irradiation had been reported to upregulate CXCL12, an effect that was reportedly further enhanced by hypoxia [28]. Consistently, we demonstrate that total body irradiation resulted in a downregulation of the miR-25-93-106b cluster followed by an increase in CXCL12.

An important target population for CXCL12-mediated recruitment and signaling are CXCR4+ cancer stem cells, which are mostly found in the invasive front and are essential for metastasis [3, 29]. The BM stromal niche provides a favorable environment for circulating tumor settlement and growth, in which CXCL12 plays a critical role in tumor cell recruitment and lodgment. CXCL12 neutralization *in vivo* within BM niches renders the BM less receptive and thus reduced homing, growth, and disease progression as shown for multiple myeloma cells [30]. Specifically, Nalm-6 pre-B acute lymphoblastic leukemia cells (ALL) have been reported to metastasize to CXCL12^+^ vascular niches in the BM due to their pronounced sensitivity to the chemoattractive cues of CXCL12 [31]. Using CXCL12-sensitive Nalm-6 cells to detect changes in the BM CXCL12 gradient, we demonstrated that the regulation of CXCL12 in the BM stromal niche via the miR-25-93-106b cluster also affected homing, and thereby metastasis. These findings should have implications for other cancers as CXCL12/CXCR4 signaling has been demonstrated as a driving mechanism for many cancers including pancreatic cancer [3, 4].

Next, we found that miR-93/106b also regulated CD274, e.g. in response to irradiation. The latter is not only used as total body irradiation for BM transplantation but also as radiotherapy for the treatment of primary and metastatic cancer. Paradoxically, our data suggest that ionizing irradiation may induce multiple resistance mechanisms that could facilitate tumor relapse, e.g. via enhancing CXCL12 and also CD274/CD279 (PD-L1/PD-1) signaling, thus limiting anti-tumor immunity [32, 33]. Specifically, cancer cells and MDSCs in the tumor microenvironment upregulate PD-L1 (CD274) to suppress anti-tumor immunity by inhibition of the T cell-mediated cytotoxicity [32, 33]. MDSCs are categorized as a group of immature monocytic and granulocytic myeloid-derived suppressor cells, in which hypoxia has been demonstrated to upregulate CD274 via hypoxia-inducible factor-1 (HIF-1). Blockade of CD274 diminished MDSC-mediated T cell suppression by modulation of the MDSC-produced cytokines [34]. To date, however, only a few studies have investigated the regulation of CD274 by miR, e.g. a study showing the role of miR-513 in modulating CD274 in human cholangiocytes [35]. Another study determined that miR-197 is downregulated in chemoresistant non-small-cell lung cancer, and restoring miR-197 using miR mimics re-sensitizes CD274^high^ drug-resistant cells to chemotherapy [36]. Our data now demonstrate that expression of CD274 in MDSC is regulated by the miR-25-93-106b cluster and its expression can be reduced significantly by the use of miR-93 mimics. It is important to note, however, that the contribution of CD274 expression on non-tumor cells to inhibit the anti-tumor response has at least been questioned[37] and recent studies in pre-clinical lung cancer models also suggested a more dominant role for cancer cell-derived CD274 [38]. It has been reported that CD274 is induced on murine melanoma cells upon communication with BM-derived CD11b^+^ cells BM-derived immune cells [39].

Consistently, we found that medium conditioned by macrophages as the most abundant and pro-tumorigenic cell population in the tumor microenvironment [20], further enhanced CD274 expression in pancreatic cancer cells. We used pancreatic cancer cells as a role model of a disease with very strong immunosuppressive properties [40]. While many human cancers such as advanced melanoma showed impressive response to treatment with immune checkpoint inhibitors, patients with pancreatic cancer did not respond to immunological checkpoint antagonists, although cancer cell-specific CD8^+^ T cells were certainly present [41, 42]. However, after depletion of fibroblast activation protein (FAP)^+^ stromal cells producing CXCL12 in the tumor, immune control of PDAC growth could be achieved by the synergistic action of a CXCL12 receptor chemokine (C-X-C motif) 4 inhibitor and anti-PD-L1 [43], an approach that is currently tested in a clinical Phase 2 trial for patients with metastatic pancreatic cancer. Here, our data suggest that expression of CD274 in pancreatic cancer cells and the surrounding immunosuppressive MDSC is inhibited by miR-93 and 106b. Therefore, our work identifies a crucial dual role for the miR-25-93-106b cluster in the regulation of both CXCL12 and CD274, thereby mimicking the synergistic action of CXCL12 and PD-L1 inhibition, and suggests therapeutic potential for therapy-resistant PDAC.

While the clinical translation of our finding based on the use of miR mimics to modulate CXCL12 and PD-L1 may be considered challenging, we have been able to enhance the expression of the miR-25-93-106b cluster using a clinical grade BET inhibitor, i.e. OTX015. This molecule reversibly binds the BET proteins BRD2, BRD3, BRD4, and BRDT, and prevents protein-protein interactions between BET proteins and acetylated histones and transcription factors. BET inhibitors attenuate cell growth and survival in several hematologic cancer models [44], but also in pancreatic cancer models [45], at least in part through the down-regulation of the critical oncogene MYC. However, the mode of action of BET inhibitors is multifaceted, cell type-dependent, and may involve a broad signaling and transcriptional rewiring of both tumor cells and the tumor stroma. Here we demonstrate for the first time that BET inhibitors are able to induce miR-93/106b in the stromal microenvironment that could be important for developing more effective combination treatments. Indeed, pancreatic cancer patients have been shown to be mostly resistant to PD-1 inhibition [46] and also BET inhibitors alone only transiently slowed tumor progression [47]. Based on the data presented herein, it will be interesting to now study the effects of PD-1 inhibitors in combination with BET inhibitors in order to enhance the efficacy of checkpoint targeting.

## MATERIALS AND METHODS

### miRNA expression array

Total RNA was fluorescently labeled (Sham: Hy3; ischemia: Hy5) and hybridized to topic-defined PIQOR™ miRXplore Microarrays. Fluorescence was detected using a laser scanner from Agilent (Agilent Technologies). Our data represent the average values for 10 mice per group. Data were subsequently ranked for average miRNA expression.

### Flow cytometry

CD45^–^ BM stromal cells were stained using anti-mouse CD45-APC, CD45-FITC, CD45-PE, SCA-1-PE (all BD Biosciences), CD140a (PDGFRa)-PECy7, and CD274-APC (both Biolegend). Myeloid cells were identified using CD11b-FITC (Biolegend) or CD11b-PerCP-Cy5.5 (eBiosciences). MDSC were stained using CD11b-PerCP Cy5.5, Gr-1-PECy7 (eBiosciences), k and LyC-Alexa700 (Biolegend). For intracellular staining of CXCL12, cells were fixed with 4% paraformaldehyde, permeabilized in 0.1% Triton X (PBS-T), and stained with SDF-1-Fluorescein (R&D; IC350F, 1:10). For surface staining, antibodies were used at 1:50 in PBS for 10min at room temperature and live cells negative for 4′,6-Diamidino-2-phenylindol (DAPI; Sigma: 1μg/ml) were analyzed on the Fortessa or sorted with the ARIA II cell sorter (both BD).

### RT-qPCR

Total RNA was isolated using QIAzol (Qiagen) and complementary DNA synthesized (for miRNA: NCode VILO miRNA cDNA synthesis kit; for non-miRNA: SuperScript VILO cDNA synthesis kit; both Invitrogen). MiR qPCR was performed using Express SYBR GreenER Supermix with premixed ROX (Invitrogen). SNORD95 was used as housekeeping gene for miR to normalize the Ct values (ΔCt), and relative expression was calculated using the 2^−ΔΔCt^ method. Primers for miRNA qPCR for miR-25, miR-93, miR-106b, and SNORD95 were purchased from Qiagen. For mRNA, QPCR was performed using PerfeCTa SYBR Green fastMix low Rox (Quanta Biosciences). Ct values were normalized to housekeeping genes such as HPRT, GAPDH, and Rps29 (ΔCt), and relative expression was calculated using the 2^−ΔΔCt^ method. The list of primers can be found in Supplementary Table 1.

### *In vivo* experiments

Unilateral hind limb ischemia was induced by ligating the deep and superficial femoral artery with an electrocoagulator and validated using this *In Vivo* Imaging System IVIS-200 as described previously [48]. Myocardial infarctions were created by ligating the left anterior descending coronary artery. Scar size was quantified with ImageJ (NIH) using the midline method [49]. BM transplantation was carried out as previously described [50]. Briefly, recipient mice were irradiated with 12Gy total body irradiation given in a single dose. Twenty-four hours later, donor BM cells were intravenously administered by retro-orbital plexus injection. Mice constitutively expressing the far-red fluorescent protein Katushka were a kind gift of Sagrario Ortega (CNIO). For *in vivo* homing analysis, recipient mice were irradiated with 12Gy and treated with 80mg/kg CyclosporineA i.p. 24h prior to the i.v. injection of 2×10^6^ DiD-labeled donor cells [51]. Mice were sacrificed 6 or 48hrs later for flow cytometry of the BM [52]. All animal procedures were conducted in accordance to the 3Rs and were approved by the Institute's Institutional Animal Care and Use Committee (CBA 68_2013 & CBA 25_2009 & PPL70-8129).

### miRNA inhibitors/mimics

MiR were inhibited using mmu-miR-93 antagomiRs (5′ → 3′: C*U*A CCU GCA CGA ACA GCA C*U*U *U*G, *phosphorothiates), mmu-miR-106b antagomiRs (A*U*C UGC ACU GUC AGC AC*U *U*U*A), or scrambled control (A*A*G *C*AC GCG CGU UGA GA*A *U*U*G) at 1.2μM (Biospring). MiR-93, miR-106b mimics, or control mimics were used at 200pmol/24-well). To induce miR106 knockdown, cells were transduced with a lentiviral construct for miR106b knockdown identified by expression of GFP (miR106b OFF GFP; LentimiRa-Off-mmu-miR-106b-5p Vector, Applied Biological Materials, Richmond, Canada).

### Invasion/migration assays

First, CD45^–^ WT BM cells or MSC were seeded into the lower wells in in 200μl RPMI medium + 2% B-27 supplement (Thermo Fisher Scientific), grown to 80% confluency, and treated with antagomirs for 6hrs. BD Matrigel™-precoated inserts, 8μm pore size, were seeded with 5×10^4^ BM or Nalm-6 cells using the same medium in the absence or presence of AMD3465 (10μM; Tocris Bioscience), inserted into above wells, and allowed to transmigrate for 42hrs. Transmigrated cells were fixed with 4 % paraformaldehyde, stained with DAPI (1:2,500), imaged by confocal microscopy, and images were analyzed using Imaris 8.0 software (Bitplane) applying the Imaris spot detection algorithm to assign a spot for each fluorescent intensity of a single nucleus. For migration studies, cells were labeled with the far red cell tracker DiD (Vybrant™ cell-labeling solution; ThermoFisher Scientific). 6-channel μ-slide (μ-slide VI ^0.4^; ibidi) were pre-filled with complete RPMI + 2% FCS + 10μM cyclosporine A (Sigma, to prevent xenogeneic response). One side was loaded with femora and tibiae fragments from mice irradiated with 12Gy, whereas the other side was loaded with 10^4^ DiD^+^ Nalm-6 cells. The number of cells that passed beyond mid-way of the channel by 20h were scored as migrating cells. In a modification of this assay, we used 1×10^5^ miR-106b OFF PDAC cells with or without CXCL12 CRISPR/Cas9 knockout as target cells and allowed 1×10^3^ (low number) Nalm-6 cells or 1×10^5^ (high number) Nalm-6 cells to migrate towards the target cells for 20h.

### Primary PDAC cultures

Pancreatic tumors were obtained from murine pancreatic cancer models (Ela-KRAS and KPC) or patient-derived xenografts as described previously[53]. Tumors were homogenized using a gentleMACS dissociator followed by enzymatic digestion with collagenase P for 15min at 37°C, and cultured in DMEM + 10% FCS. Outgrowing epithelial clones were then further expanded to heterogeneous cancer cell cultures. Stromal cells were mechanically removed by cell scraping and cultured separately. For some experiments, we required murine stromal cell rich primary PDAC cultures and used the stroma rich CKT111 PDAC cultures with mesenchymal features. Culture under low adhesion conditions to enrich for cancer stem cells and human pancreatic cancer xenografts have been described previously [54]. In some experiments, cells were treated with the BET inhibitor OTX015 (Cayman Chemicals; 500nM). To culture spheres enriched in cancer stem cells, cells were re-suspended in 1X DMEM/F-12 (Gibco™) supplemented with 20ng/ml FGF-2 (CellGS), 0.4% Amphotericin B, 1% Penicillin/Streptomycin, 2% B27 supplement (Gibco™) and 200mM of L-glutamine (Gibco™). A cell suspension of 10,000cells/ml was then prepared and distributed into ultra-low attachment surface flasks (Corning, NY, NY) for one week. Prior to use, spheres were filtered (40 μM for human spheres, 20 μM cell strainer for murine spheres).

### Generation of Cxcl12 knock-out cells

Cxcl12 knock-out clones were generated using CRISPR/Cas9 gene editing as previously described [55]. Briefly, 4 sgRNAs targeting exon 2 of mouse Cxcl12 were cloned into the lentiCRISPR v2 backbone (a gift from Feng Zhang; Addgene plasmid # 52961) and transfected into CKT111 cells. Single cell-derived clones were generated following puromycin selection and Cxcl12 knock-out was validated by ELISA for murine CXCL12 (Duoset, R&D) from cell culture supernatants. We used the following targeting sgRNA sequences: sg#1 TGAGCTACCGATGCCCCTGC; sg#2 CAGA TGCTTGACGTTGGCTC; sg#3 GATTTTCAGATGC TTGACGT; sg#4 TGACGTTGGCTCTGGCGATG

.

### 3′UTR luciferase reporter assays

The 3′UTR-*Cxcl12* Gaussia luciferase reporter construct (2μg per 24-well) and a control 3′UTR-reporter construct (all from Genecopoeia) were transfected into HEK293T cells using Lipofectamine (Invitrogen). Co-transfection per 24-well (containing 5×10^4^ cells) was performed with double-stranded miR-93 mimic, miR-106b mimic, or control mimic (Sigma; all at 200pmol/24-well). In addition, a Renilla Luciferase reporter construct was used as a transfection efficiency control for normalization (0.6 μg per 24-well). Secreted Gaussia luciferase activity was measured using the Secrete-Pair^®^ Dual Luminescence Assay Kit (Genecopoeia). Renilla luciferase activity was measured using the Dual-Luciferase^®^ Reporter Assay System (Promega).

### Immunostaining

For the live confocal microscopy of migrated DiD^+^ cells, nuclei were counterstained with Hoechst (Hoechst 33342; 1μg/ml), and the bones (sternum) placed into an Attofluor ™ cell chamber (Life Technologies) for viewing live specimens prior to analysis using a Leica SP5 confocal microscope for the presence of DiD^+^ Nalm-6 cells. For the confocal analysis of CXCL12 expression in the BM of WT and Mir-25-93-106b-KO bone fragments, we fixed the bones in 4% paraformaldehyde for 10min at room temperature, washed twice with PBS, incubated in PBS + 0.1% Triton X (PBS-T) for 15min at room temperature, followed by incubation of the CXCL12-Fluorescein (R&D; IC350F) antibody 1:10 in PBS-T for 30min at room temperature. Nuclei were counterstained with DAPI (Sigma, 1μg/ml) prior to analysis using a Leica SP5 confocal microscope.

### Immunohistochemistry

Bone fragments from sternum were harvested, fixed in 10% neutral buffered formalin, decalcified, and then paraffin embedded. Immunohistochemical staining on 2.5μM sections was carried out using the Ventana Discovery XT system (Roche). Sections were deparaffinized, hydrated, and loaded on the Discovery XT. Antigen retrieval was performed using citrate buffer solution. Endogenous peroxidases were quenched using H_2_O_2_ reagent (Ventana). Primary antibodies against myeloperoxidase and factor VIII (both Dako, rabbit polyclonal, diluted 1:300), and CD31 (Abcam; rabbit polyclonal, diluted 1:50), and CD274 (Cell Signaling; diluted 1:150, rabbit mAb recognizing endogenous levels of total PD-L1 protein), were incubated for 20min and detected using an anti-rabbit secondary antibody and the ChromaMap DAB detection kit (Ventana). Tissues were counterstained with hematoxylin and image analysis was performed using Pannoramic Viewer Software (3DHISTECH).

### miR *in situ* hybridization (ISH)

ISH for miR-106b was performed as previously described [56]. Briefly, FFPE sections at 5μm thickness were deparaffinised, rehydrated and boiled with 0.01M sodium citrate buffer (pH 6) for 10 minutes. The samples were then prehybridised for 20 minutes at 48°C, before incubating with 25nM DIG-labelled LNA oligonucleotide probe (Exiqon) for 1 hour at 48°C. Samples were blocked with 5% sheep serum for 1 hour at room temperature, then incubated with alkaline phosphatase-conjugated anti-DIG antibody (1:1,000) overnight at 4°C. Slides were developed with NBT/BCIP for 30min, then lightly counterstained with methyl green before dehydration and mounting.

### Statistical analysis

Unless stated otherwise, results are expressed as the means ± SEM. Statistical analyzes were performed with SPSS 22.0 (San Diego, CA) comparing continuous variables by non-parametrical Mann-Whitney U and Kruskal-Wallis tests. Statistical analysis of Kaplan-Meier curves is performed by the log-rank test. The significance is given as p<0.05.

## SUPPLEMENTARY MATERIALS FIGURES AND TABLES


