# Supplementary material for: The miR-25-93-106b cluster regulates tumor metastasis and immune evasion via modulation of CXCL12 and PD-L1
Source: Oncotarget. 2017 Feb 17;8(13):21609–25. doi: 10.18632/oncotarget.15450 (PMC5400610; doi:10.18632/oncotarget.15450)
Supplement: Supplementary file 1 [file oncotarget-08-21609-s001.pdf]

The miR-25-93-106b cluster regulates tumor metastasis and immune evasion via modulation of CXCL12 and PD-L1

SUPPLEMENTARY FIGURES AND TABLE

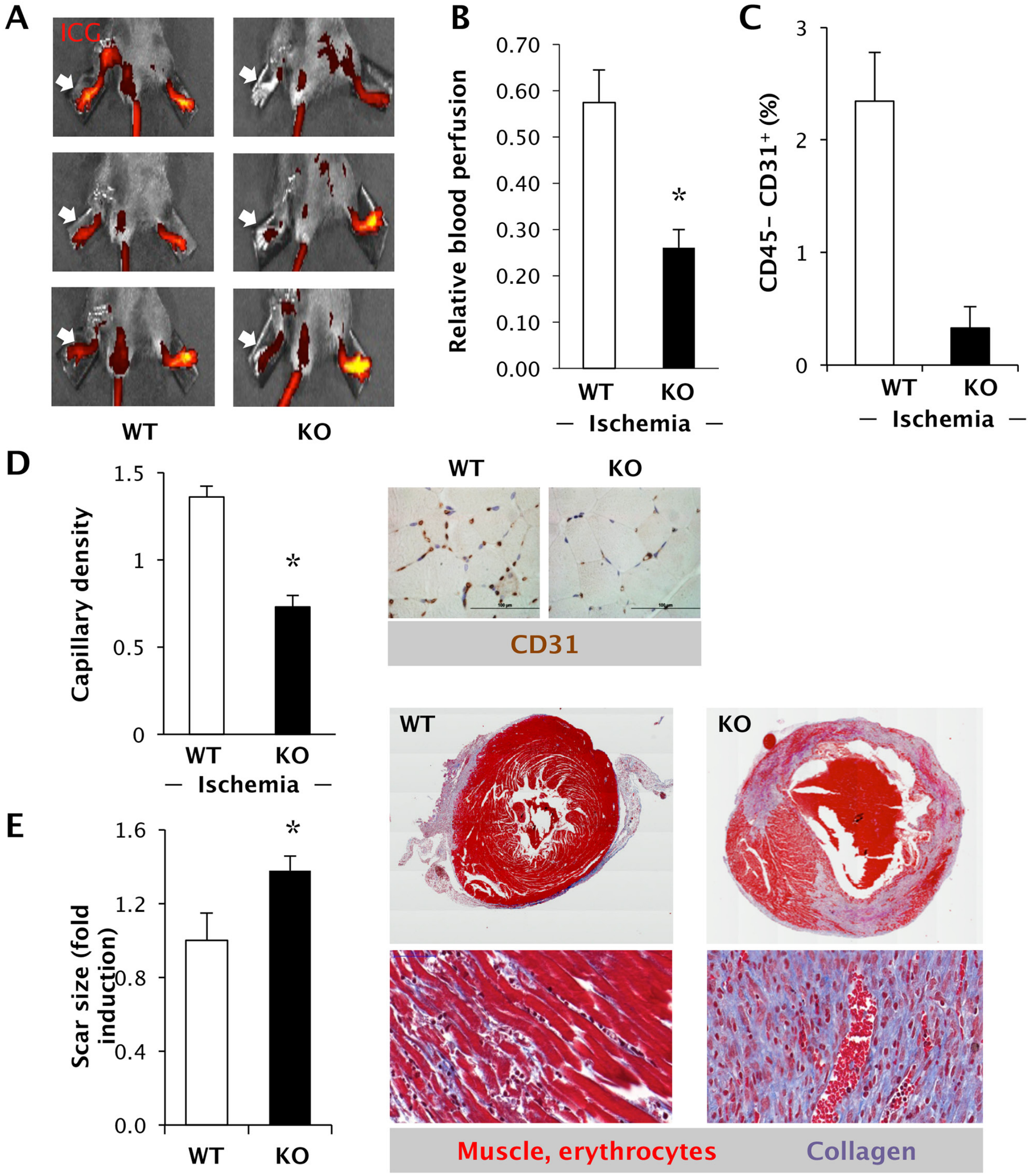

Supplementary Figure 1: Induction of hindlimb / myocardial ischemia in WT and miR-25-93-106b KO mice. A. Assessment of ischemia induction by indocyanogreen (ICG) and visualization by *in vivo* fluorescence imaging (IVIS). Arrows indicate ischemic hindlimb. **B.** Quantification of relative blood perfusion; n=6-7, * p<0.05. **C.** Quantification of $CD31^{+}CD45^{-}$ endothelial cells in homogenized ischemic muscles by flow cytometry. **D. Left:** Quantification of capillary density (number of $CD31^{+}$ capillaries/myocyte); n=12, * p<0.05. **Right**: Representative IHC for CD31 expression in cross-sections of ischemic adductor muscles. **E.** Induction of myocardial infarction in WT and mir-25-93-106b KO mice. **Left:** Quantification of myocardial scar size; n=3-4 * p<0.05. **Right**: Overview (**upper**) and higher magnification (**lower**) of transversely sectioned hearts one week after onset of infarction by Masson's trichrome stain to visualize blue collagen deposition.

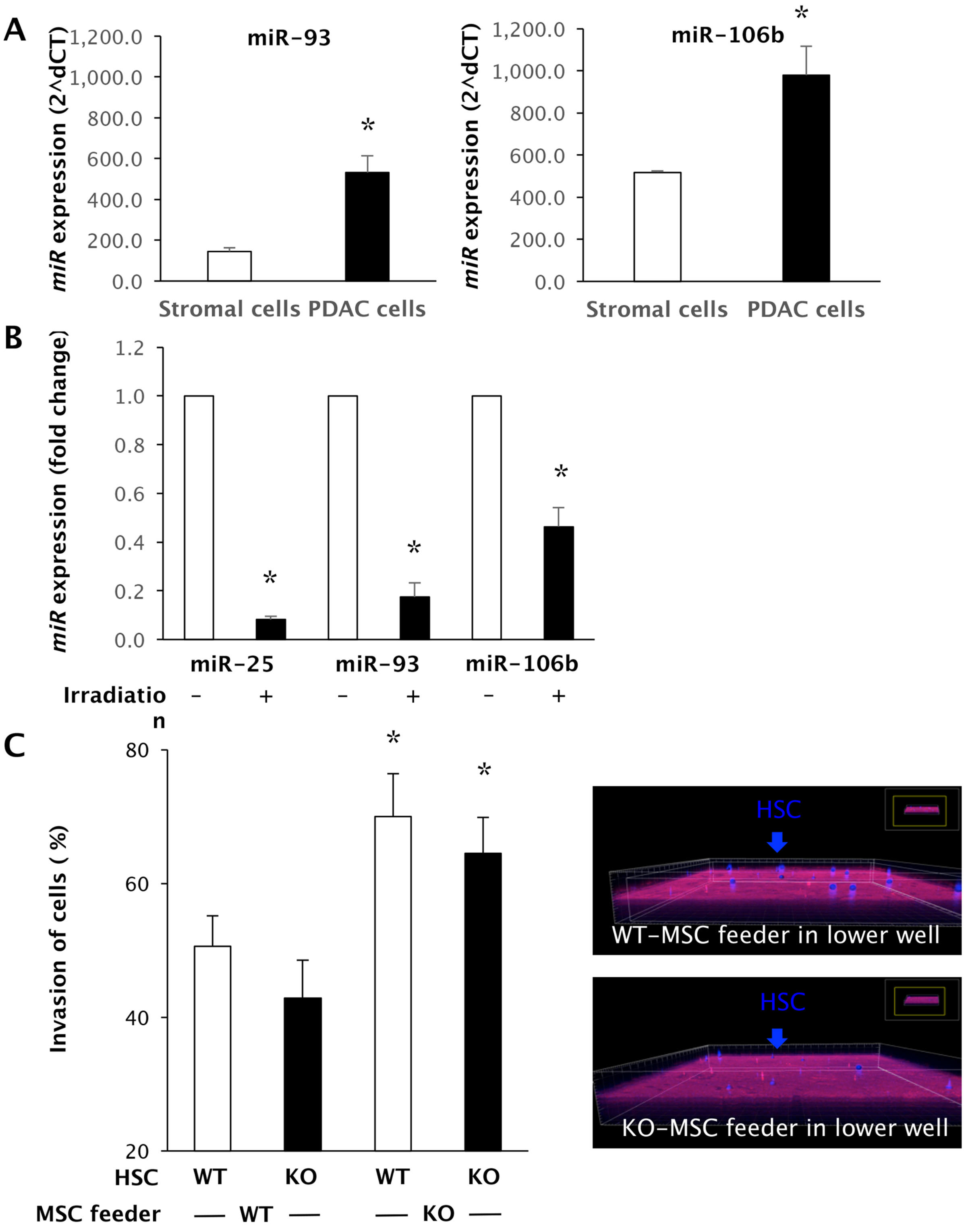

Supplementary Figure 2: Role of the miR-25-93-106b cluster in the bone marrow niche. A. *Ex*pression of members of the miR-25-93-106b cluster in primary tumour stromal cells versus cancer cells; n=4-5, *p<0.05. **B.** *In vivo* expression of members of the miR-25-93-106b cluster in BM cells with or without irradiation; n=4, *p<0.05, 6 hours following irradiation. **C.** Knockout of the miR-25-93-106b cluster in the bone marrow niche enhances invasion of hematopoietic CD45^{+}c-kit^{+} cells (HSC) *in vitro*, n=12-13, * p<0.05.

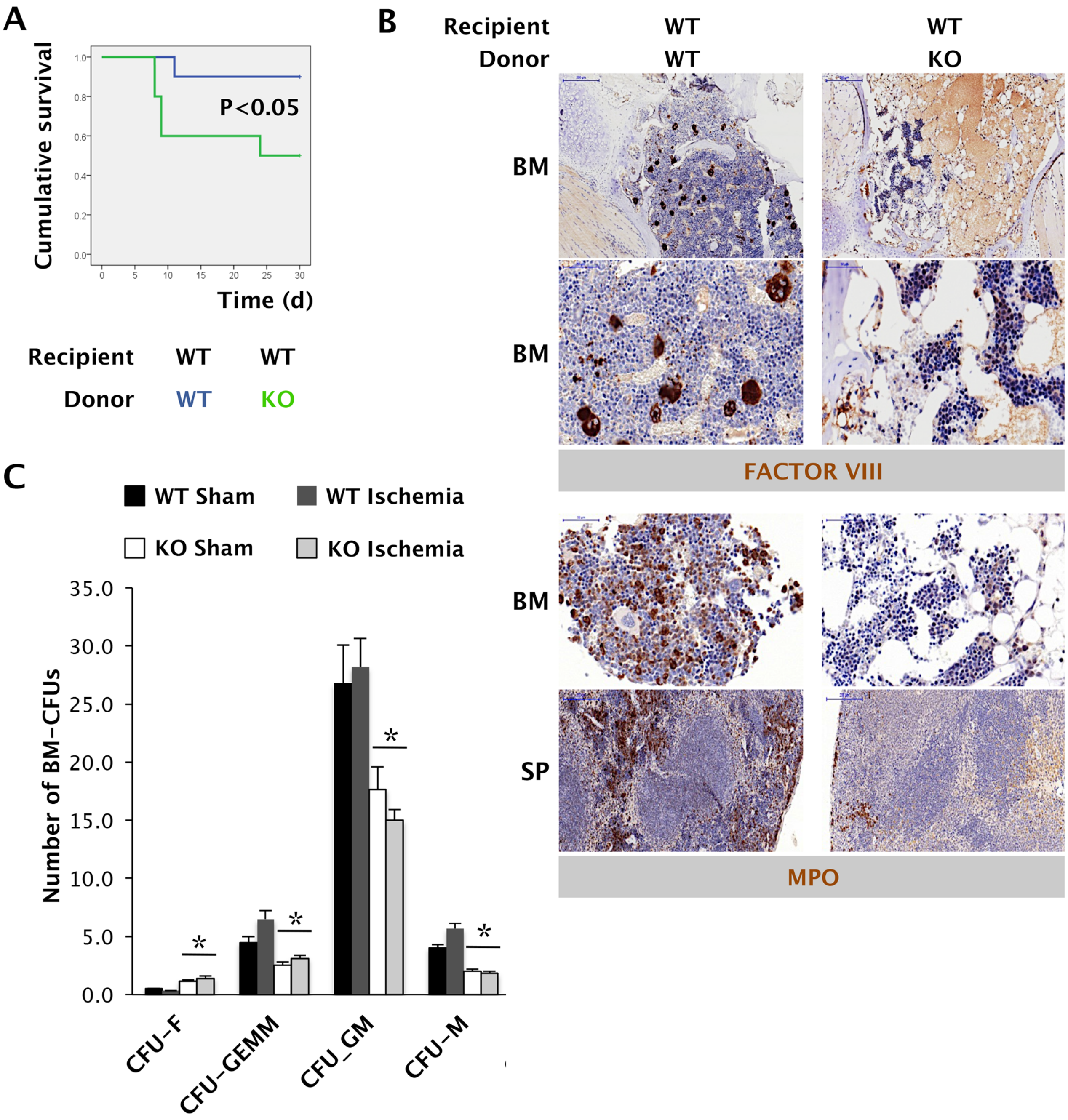

Supplementary Figure 3: Dysregulated hematopoiesis in miR-25-93-106b KO bone marrow cells after low dose transplantation of miR-25-93-106b KO vs. WT bone marrow (BM) into irradiated WT recipients. A. Kaplan-Meier curves of recipient WT mice following BM transplantation (Tx) with 100,000 miR-25-93-106b KO or WT BM cells; quantification (n=10, * $p<0.05$). **B.** Representative IHC of BM sternal segments after BM Tx. Upper panel: Staining for factor VIII (FVIII) identifying cells of the megakaryocyte lineage. Low (top) and high (bottom) magnification. Lower panel: Staining for myeloperoxidase (MPO) identifying neutrophilic granulocytes in the BM (top) and in the spleen (SP, bottom). **C.** Colony-forming units (CFU) based on 10^3 $CD45^+$c-KIT^+ HSC derived from mir-25-93-106b KO or WT BM following induction of ischemia or sham treatment. CFU-fibroblasts (F), CFU-granulocyte, erythroid, macrophage, megakaryocyte (GEMM), CFU-granulocyte, macrophage (GM), and CFU-macrophage (M) were scored; n=5-6, * $p<0.05$.

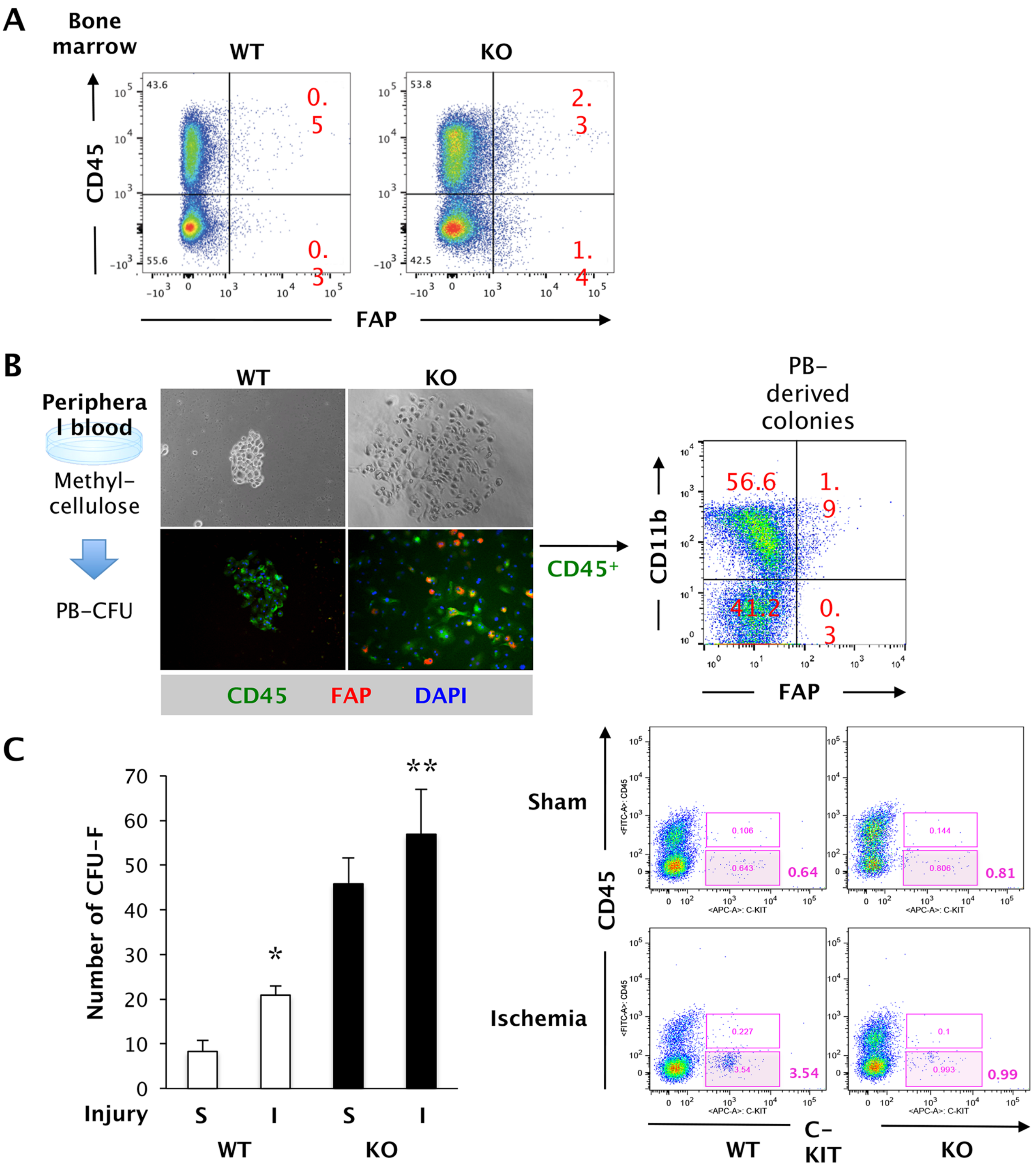

Supplementary Figure 4: Fibroblast and fibrocyte progenitors in the bone marrow and peripheral blood of miR-25-93-106b KO and WT mice. A. Flow cytometry of BM CD45$^-$FAP$^+$ fibroblasts and CD45$^+$FAP$^+$ fibrocytes. **B. Left:** Adherent flat fibroblast/fibrocyte colonies (CFU-F) derived from 100µl of lysed peripheral blood grown in methylcellulose. Bright field images indicating the colony sizes in miR-25-93-106b KO and WT mice (**top**). Immunostaining of CFU-F (**bottom**). **Right:** flow cytometry of CD45$^+$ peripheral blood-derived colonies. **C.** Quantification of peripheral blood CFU-F (**left**) and subsequent analysis by flow cytometry (**right)** following induction of ischemia (I) or sham (S) treatment; n=7, * p<0.05 for WT-I vs WT-S; ** p<0.05 for miR-KO-S vs WT-S.

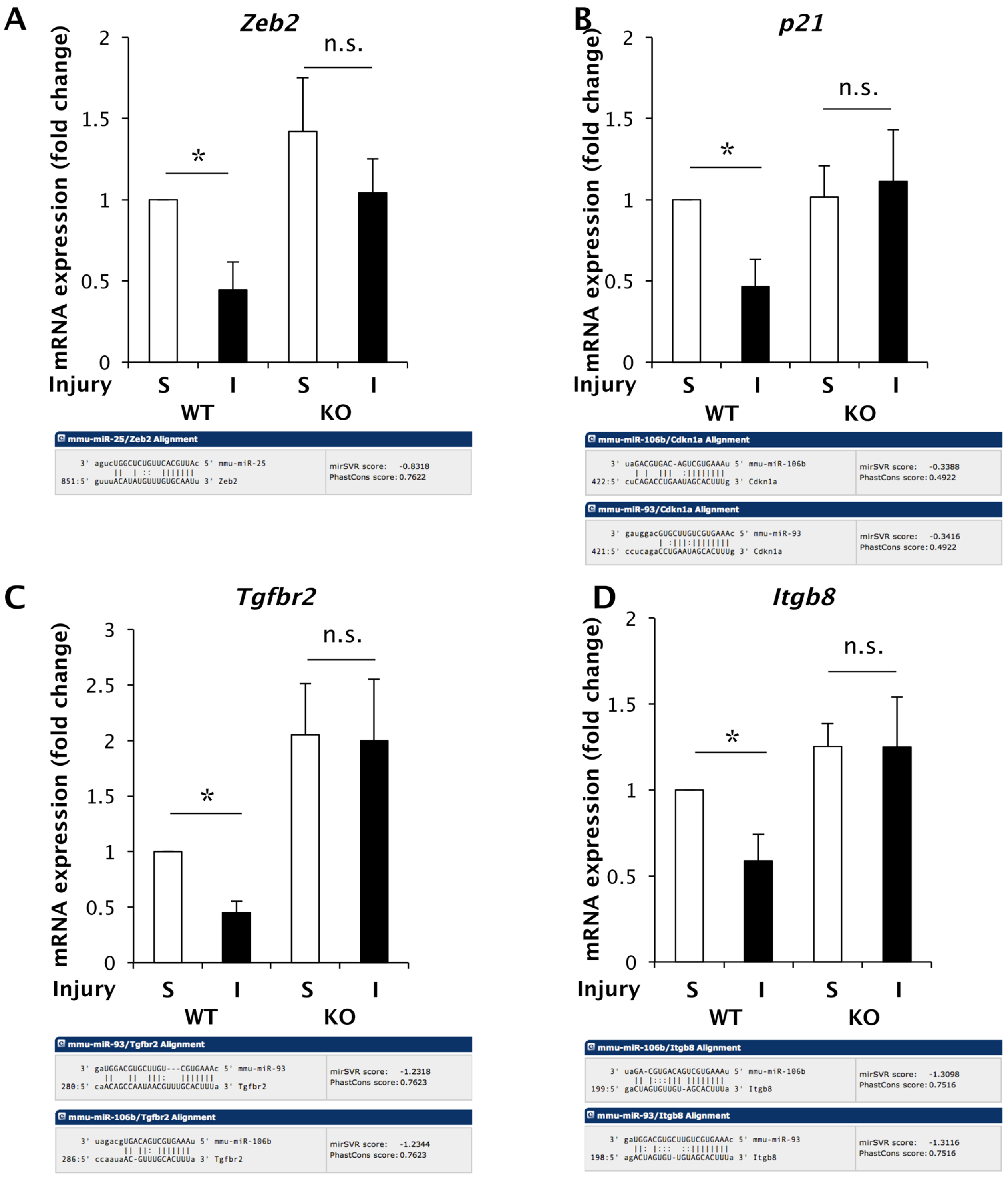

Supplementary Figure 5: Regulation of miR-25-93-106b cluster targets in the BM stromal niche *in vivo*. $CD45^-$ BM cells from miR-25-93-106b$^{-/-}$ (KO) vs WT mice following induction of ischemia (I) or sham (S) treatment for 48 hours were sorted and analyzed for established and putative target genes. The *in silico* prediction for the selected target genes is depicted underneath the quantification. Expression of **A.** *Zeb2*, **B.** *p21 (Cdkn1a)*, **C.** *Tgfbr2*, and **D.** *Itgb8* in $CD45^-$ BM cells; n=4-5, * p<0.05 for WTI vs. WTS.

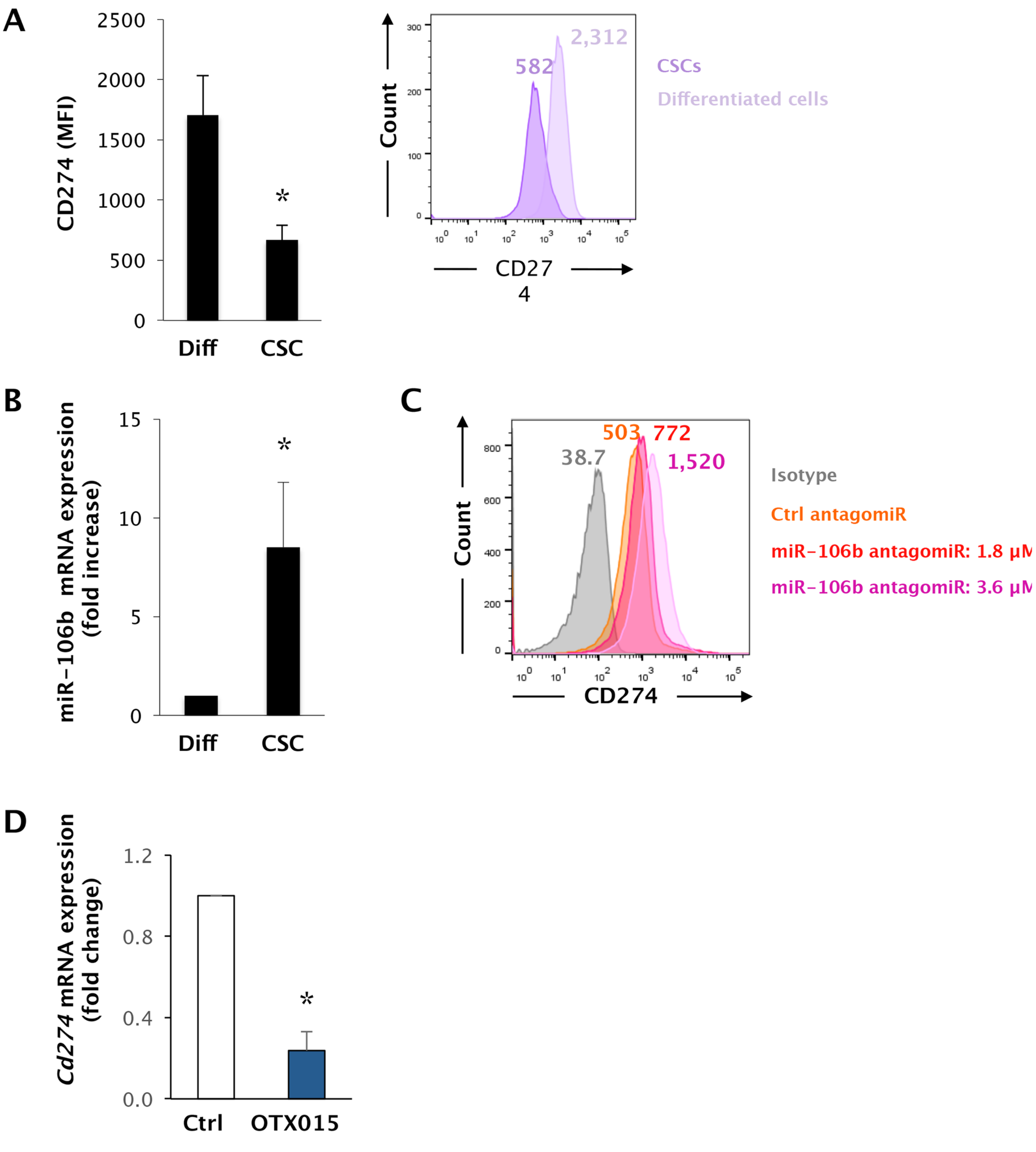

Supplementary Figure 6: Regulation of CD274 in human neoplastic cells. **A.** CD274 and **B.** inverse mir-106b expression in pancreatic cancer stem cells (CSC) and differentiated (Diff) cancer cells of human PDAC cultures; n=3, p<0.05. MFI values are displayed in the histogram. **C.** CD274 expression in human cancer cells following treatment with miR-93 and miR-106b antagomiRs for 20 h. The mean fluorescence intensity (MFI) is annotated in the histogram. **D.** Effect of treatment of primary PDAC cells with 500nM OTX for 72 hours on the target gene cd274, n=6, * p<0.05.

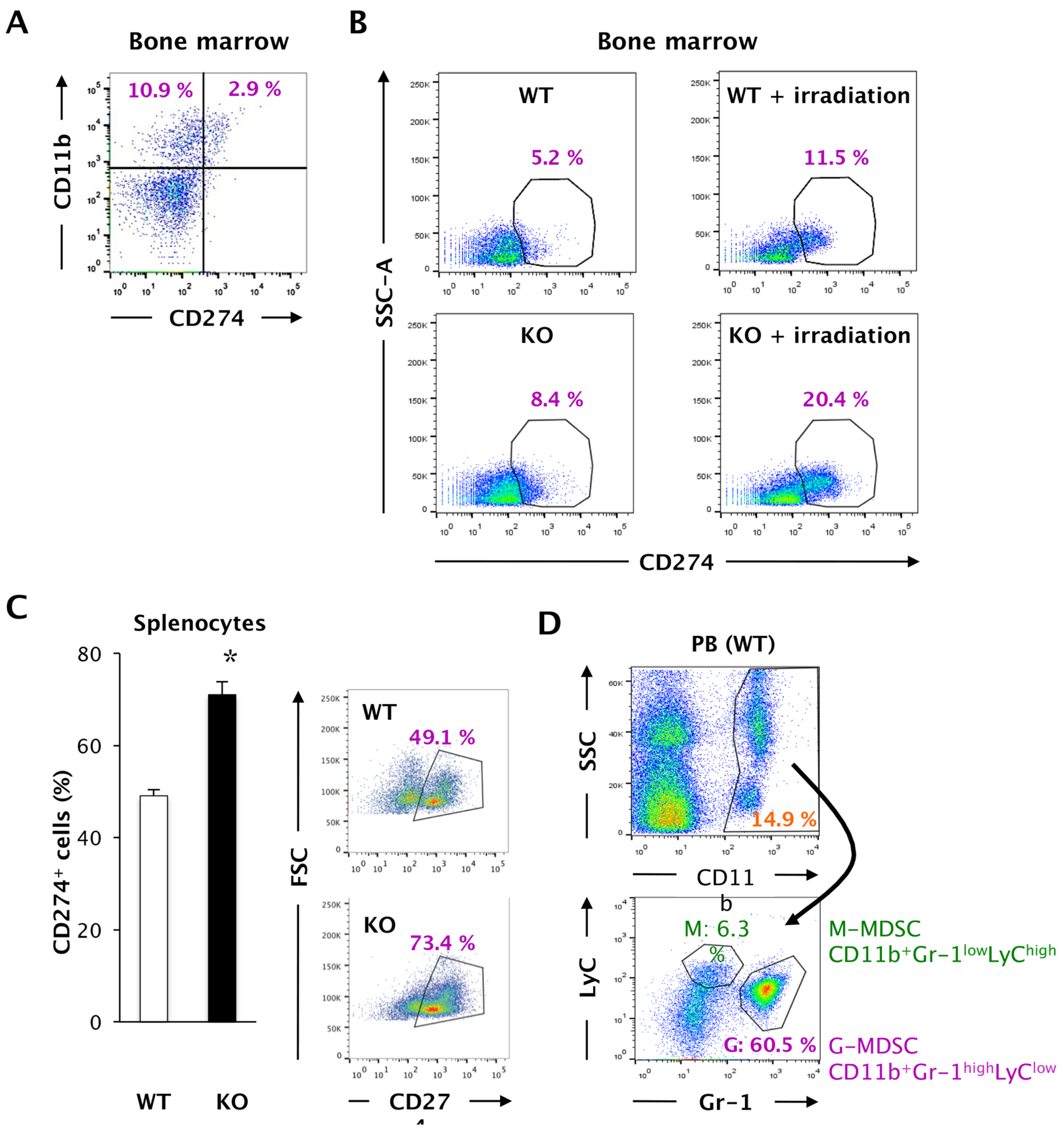

Supplementary Figure 7: Regulation of the miR-25-93-106b target CD274 in bone marrow (BM) myeloid CD11b$^+$ cells, splenocytes, and MDSC. **A.** Representative flow cytometry plots for CD274 expression in CD11b$^+$ BM cells. **B.** Representative flow cytometry of CD274 expression in BM cells 6 hours following total body irradiation. **C.** Expression of CD274 in splenocytes; quantification (left) and representative flow cytometry (right); n=4, * $p<0.05$. **D.** Flow cytometry gating strategy for M-MDSC and G-MDSC in peripheral blood. Further analysis for CD274 expression is shown in Figure 7C.

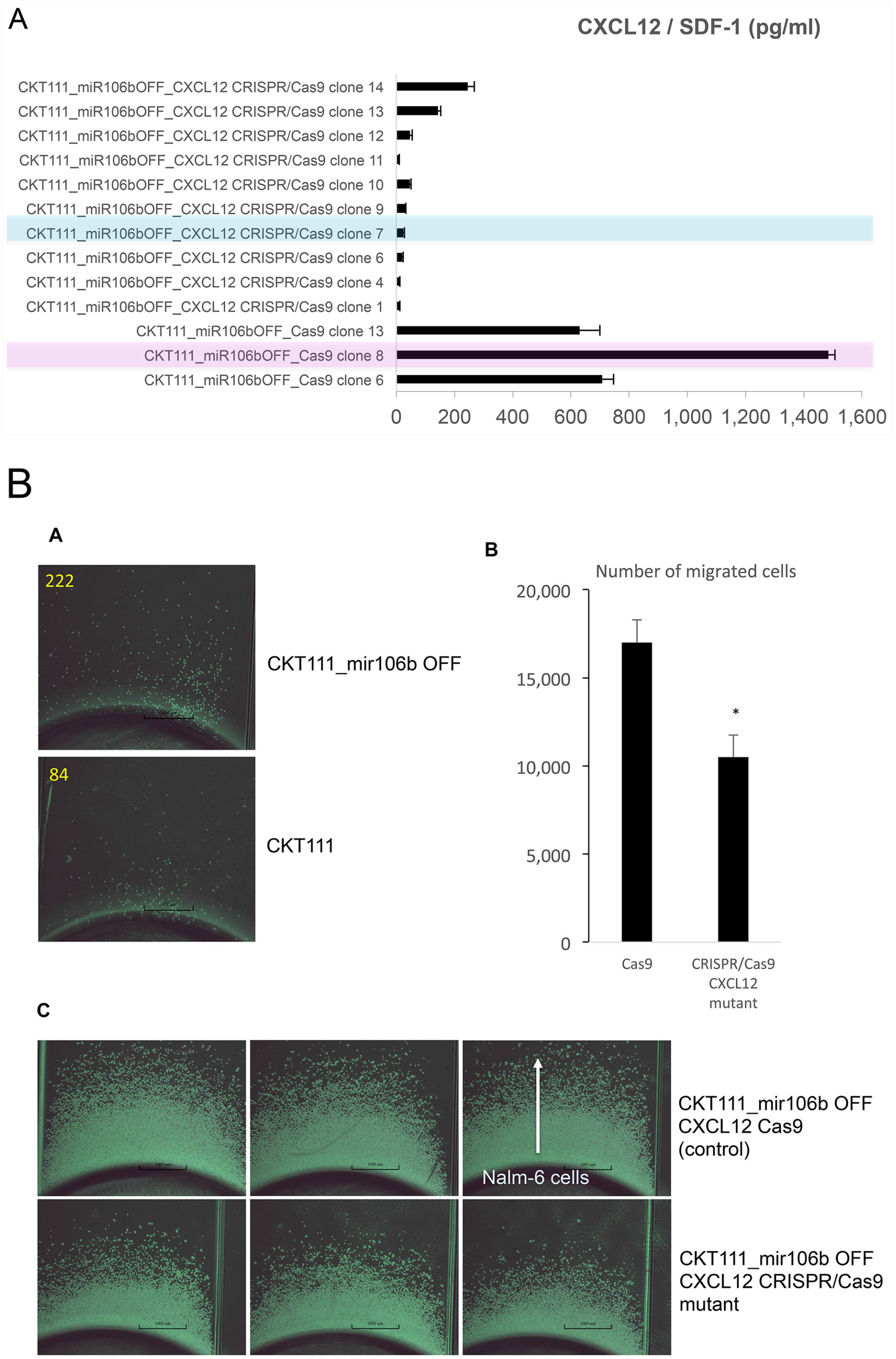

Supplementary Figure 8: Generation and validation of CXCL12-deficient miR106b OFF clones. PDAC-derived stromal cell rich CKT111 miR106b OFF cells underwent CRISPR / Cas9 gene editing to generate CXCL12 mutants deficient in the production of CXCL12 as measured by ELISA (n=3). In the next experiments we used the CXCL12-deficient clone 7 (blue) and the CXCL12-competent clone 8 (red). Migration assay using CXCL12-deficient versus CXCL12-competent miR-106 OFF cells. **A.** Migration of 1x 10^3 Nalm-6 leukemia cells towards stroma rich CKT111 PDAC cells with or without miR-106b knockdown (miR-106b OFF). The number of migrated cells are given in yellow. **B.** Quantification of the migration of 1x 10^5 Nalm-6 leukemia cells towards CXCL12-deficient or CXCL12-competent miR-106b OFF CKT111 PDAC cells located at the end of the migration channel; n=3, * $p<0.05$. **C.** Representative images for the migration of 1x 10^5 Nalm-6 leukemia cells towards CXCL12-deficient or CXCL12-competent miR-106b OFF CKT111 PDAC cells are shown. An arrow indicates the direction of Nalm-6 cell migration.

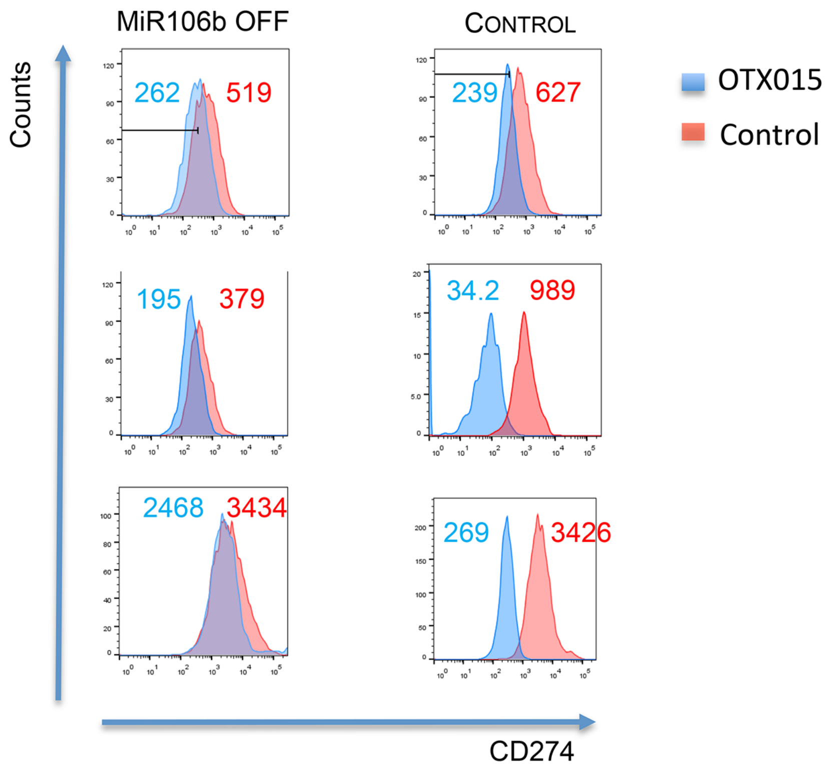

Supplementary Figure 9: Expression of CD274 in response to treatment with OTX015 in the presence or absence of miR-106b (miR-106b OFF). Different stroma rich CKT111 PDAC clones were treated with 500nM OTX015 or control (ethanol) for 72h. The marker in the top panels indicates the isotype control.

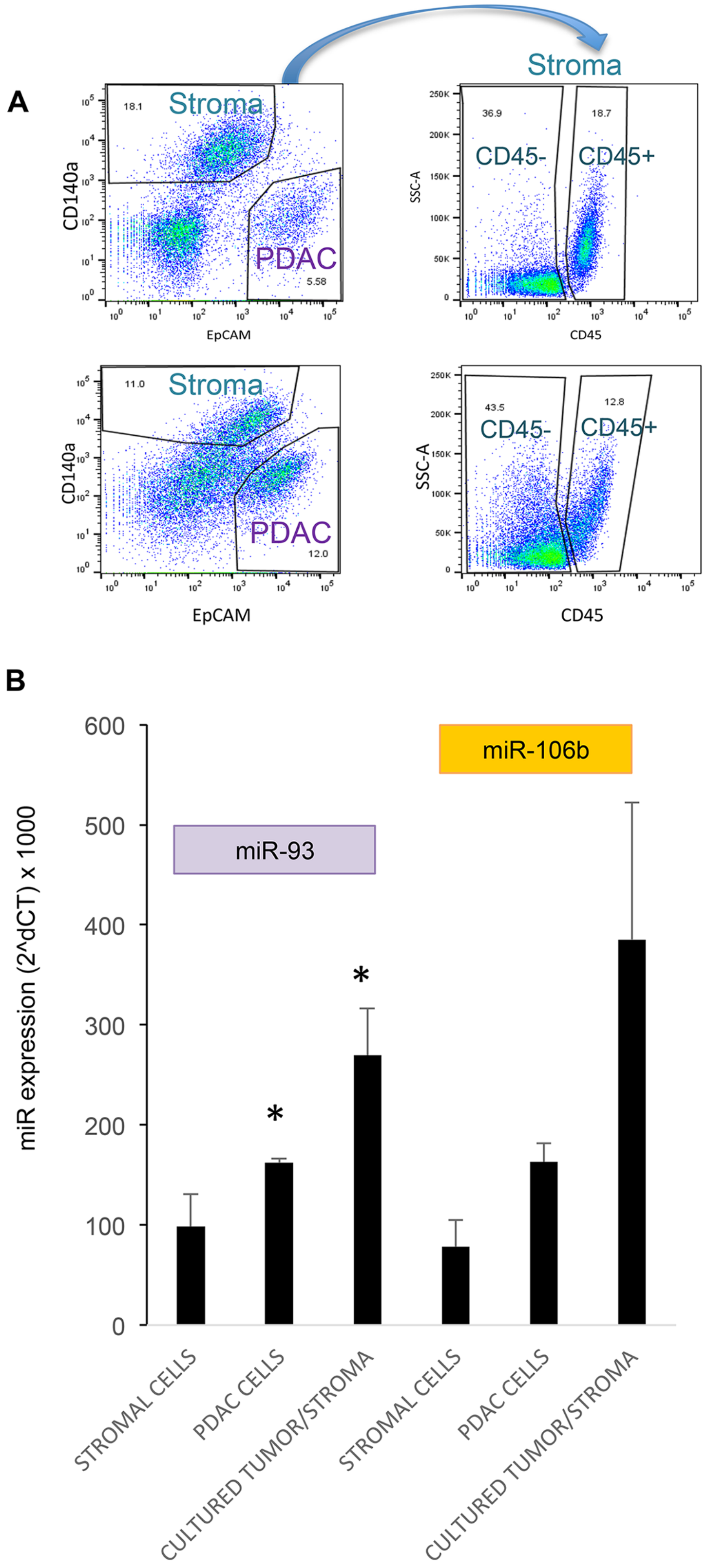

Supplementary Figure 10: Assessment of the expression of members of the miR-25-93-106b cluster in freshly sorted cancer vs stromal cells. A. Stromal cells were sorted for CD140a and further analyzed for CD45, while cancer cells were sorted for EpCAM. **B.** MiR expression was determined by qRT-PCR from three different KPC tumours (the sorting strategy is shown for two tumours; n=3, * $p<0.05$.

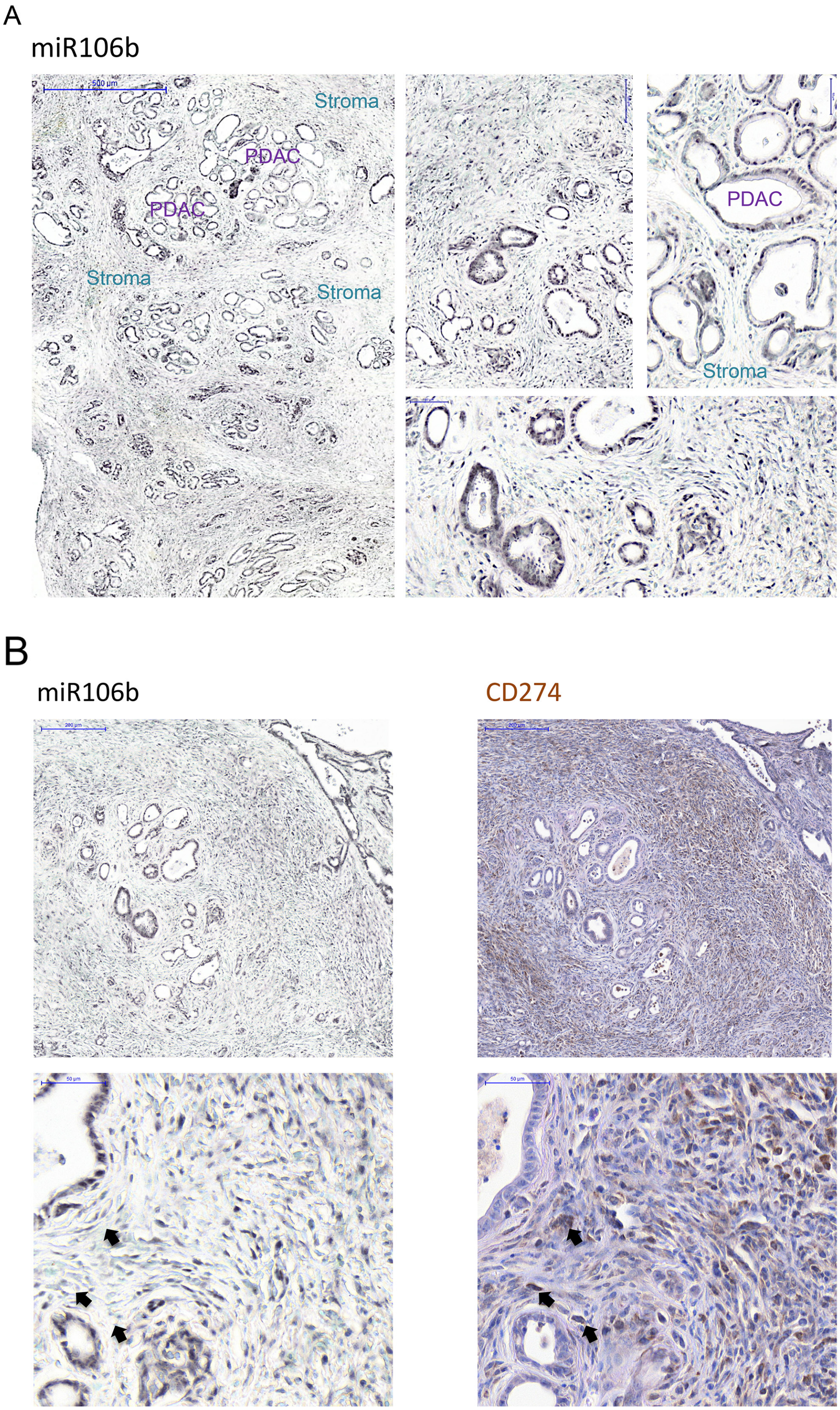

Supplementary Figure 11: A. *In situ* hybridization (ISH) for miR-106b in murine PDAC tumours. MiR-106b is expressed in both cancer and stromal compartments of the tumour. **B.** MiR-106b inversely regulates its targets in PDAC tumours. We performed ISH for miR-106b in a murine PDAC tumour. IHC for CD274 is performed on consecutive sections and shows inverse regulation mainly in the stromal cell compartment (arrows indicate examples) (*Continued*)

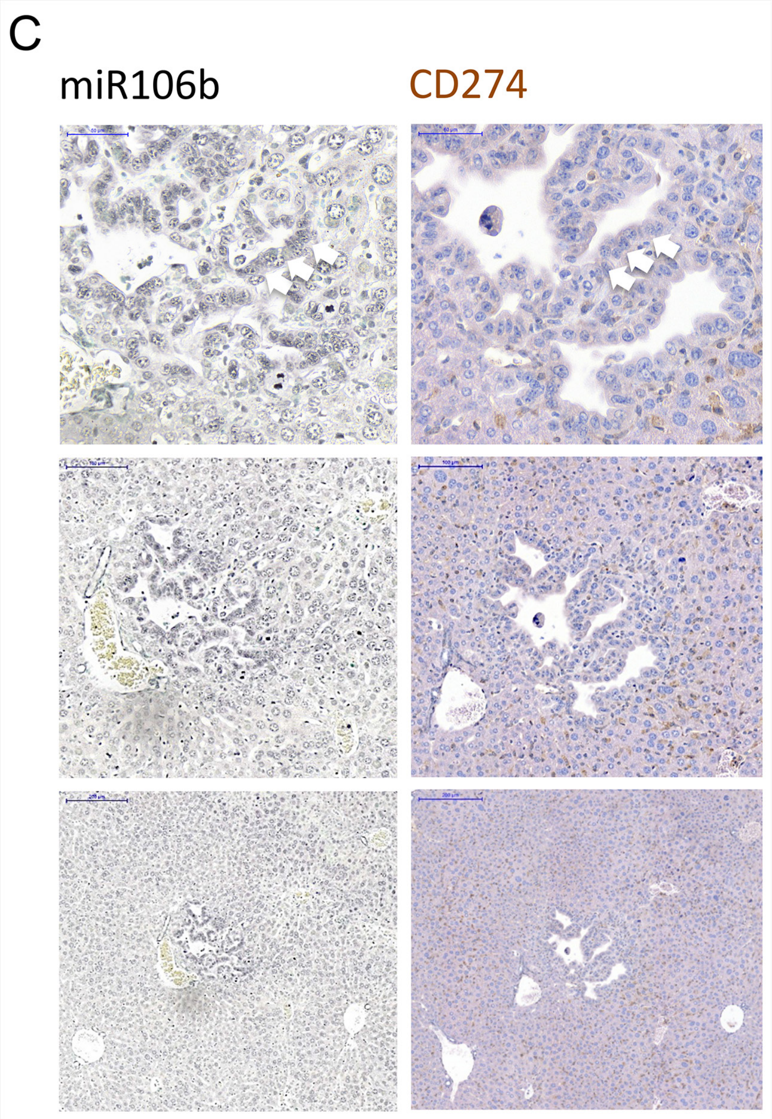

Supplementary Figure 11: (*Continued*) C. MiR-106b inversely regulates its targets in PDAC-derived liver metastasis. We performed ISH for miR-106b in murine liver metastases. IHC for CD274 is performed on consecutive sections and shows inverse regulation in the cancer cell compartment (arrows indicate examples).

Supplementary Table 1: List of utilized primers

Gene	Forward primer	Reverse primer
**29 Rps**	CGGTCTGATCCGCAAATACG	AGGTCGCTTAGTCCAACTTAAT
**HPRT**	GTTGGGCTTACCTCACTGCT	TCATCGCTAATCACGACGCT
**GAPDH**	CCGGGTTCCTATAAATACGGACTG	CCAATACGGCCAAATCCGTT
**CXCL12 (SDF-1)**	CAGATTGTTGCACGGCTGAA	CCTCGGGGGTCTACTGGAAA
**PD-L1 (CD274)**	TCTCCTCGCCTGCAGATAGT	TAAACGCCCGTAGCAAGTGA
**ZEB2**	GGCAAGGCCTTCAAGTACAA	AAGCGTTTCTTGCAGTTTGG
**P21**	ACCAGCTGTGGGGTGAGGAGG	TGCCTGTGGCACCTTTTATTCTGCT
**TgfbR2**	TCCCAAGTCGGTTAACAGTGA	TGCAGGACTTCTGGTTGTCG
**ITGB8**	GTGTGCTGGGCATGGGGAGTG	GTGCCTCTCCCGCTGCAAACT
